# An optimized bicistronic chimeric antigen receptor against GPC2 or CD276 overcomes heterogeneous expression in neuroblastoma

**DOI:** 10.1172/JCI155621

**Published:** 2022-08-15

**Authors:** Meijie Tian, Adam T. Cheuk, Jun S. Wei, Abdalla Abdelmaksoud, Hsien-Chao Chou, David Milewski, Michael C. Kelly, Young K. Song, Christopher M. Dower, Nan Li, Haiying Qin, Yong Yean Kim, Jerry T. Wu, Xinyu Wen, Mehdi Benzaoui, Katherine E. Masih, Xiaolin Wu, Zhongmei Zhang, Sherif Badr, Naomi Taylor, Brad St. Croix, Mitchell Ho, Javed Khan

**Affiliations:** 1 Genetics Branch, Center for Cancer Research, National Cancer Institute (NCI), NIH, Bethesda, Maryland, USA.; 2 Advanced Biomedical Computational Science, Frederick National Laboratory for Cancer Research, Frederick, Maryland, USA.; 3 Single Cell Analysis Facility, Center for Cancer Research, NIH, Bethesda, Maryland, USA.; 4 Mouse Cancer Genetics Program, Center for Cancer Research, NCI, Frederick, Maryland, USA.; 5 Laboratory of Molecular Biology, Center for Cancer Research and; 6 Pediatric Oncology Branch, Center for Cancer Research, NCI, NIH, Bethesda, Maryland, USA.; 7 Cancer Research UK Cambridge Institute, University of Cambridge, Cambridge, United Kingdom.; 8 Cancer Research Technology Program, Leidos Biomedical Research Inc., Frederick National Laboratory for Cancer Research, Frederick, Maryland, USA.; 9 Experimental Immunology Branch, Center for Cancer Research, NCI, NIH, Bethesda, Maryland, USA.

**Keywords:** Immunology, Therapeutics, Cancer immunotherapy

## Abstract

Chimeric antigen receptor (CAR) T cell therapies targeting single antigens have performed poorly in clinical trials for solid tumors due to heterogenous expression of tumor-associated antigens (TAAs), limited T cell persistence, and T cell exhaustion. Here, we aimed to identify optimal CARs against glypican 2 (GPC2) or CD276 (B7-H3), which were highly but heterogeneously expressed in neuroblastoma (NB), a lethal extracranial solid tumor of childhood. First, we examined CAR T cell expansion in the presence of targets by digital droplet PCR. Next, using pooled competitive optimization of CAR by cellular indexing of transcriptomes and epitopes by sequencing (CITE-Seq), termed P-COCC, we simultaneously analyzed protein and transcriptome expression of CAR T cells to identify high-activity CARs. Finally, we performed cytotoxicity assays to identify the most effective CAR against each target and combined the CARs into a bicistronic “OR” CAR (BiCisCAR). BiCisCAR T cells effectively eliminated tumor cells expressing GPC2 or CD276. Furthermore, the BiCisCAR T cells demonstrated prolonged persistence and resistance to exhaustion when compared with CARs targeting a single antigen. This study illustrated that targeting multiple TAAs with BiCisCAR may overcome heterogenous expression of target antigens in solid tumors and identified a potent, clinically relevant CAR against NB. Moreover, our multimodal approach integrating competitive expansion, P-COCC, and cytotoxicity assays is an effective strategy to identify potent CARs among a pool of candidates.

## Graphical abstract

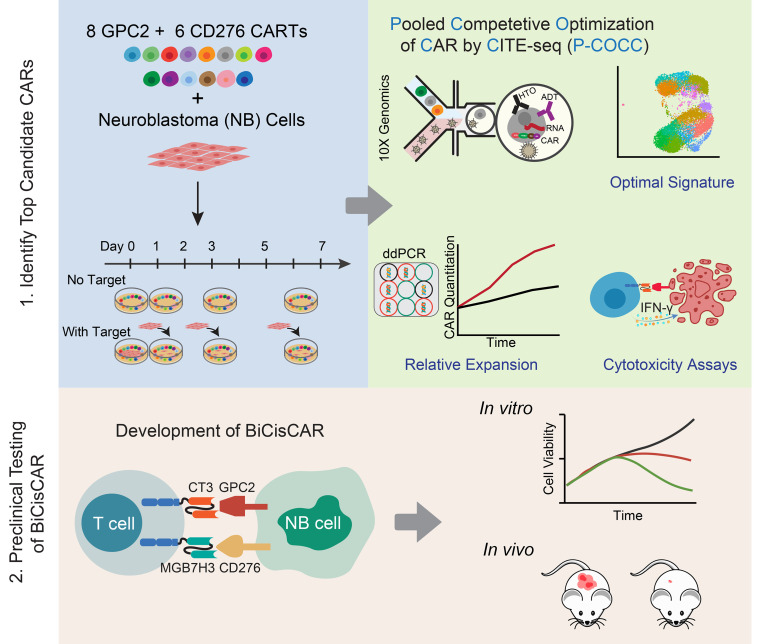

## Introduction

Pediatric neuroblastoma (NB), the most common extracranial solid cancer of childhood, generally affects children in their first decade of life (1). One of the common genetic alterations in NB is amplification of the *MYCN* oncogene (*MYCN*-A), which is associated with worse overall survival, increased metastasis, and a higher risk of disease than for patients with *MYCN*-not-amplified (*MYCN-*NA) tumors (2). Genomic studies have only identified druggable somatic mutations in less than 10 % of NBs (3), and targeting MYCN or other NB core regulatory transcription factors has remained a challenge. Recently, immunotherapy with anti-GD2 mAbs along with GM-CSF and the differentiating agent isotretinoin has shown efficacy, and it is now the standard of care for high-risk NB. Despite the recent improvements in outcomes with anti-GD2 immunotherapy, overall survival remains low at approximately 50% (4). Hence, additional therapies are needed to treat this highly aggressive cancer. Chimeric antigen receptor (CAR) T cell therapy is effective in treating refractory and relapsed B cell leukemia and lymphoma, with 6 CARs targeting CD19 or B cell maturation antigen (BCMA) approved by the FDA (5). However, these results have not been recapitulated in solid tumors, given the heterogeneous expression of tumor-associated antigens (TAAs) (6, 7), limited persistence of CAR T cells, inadequate tumor trafficking, T cell exhaustion, and a hostile tumor microenvironment (8, 9). To date, 6 NB TAAs, including disialoganglioside (GD2) (10–12), L1 cell adhesion molecule (L1-CAM; CD171) (13, 14), glypican 2 (GPC2) (6, 15, 16), CD276 (B7-H3) (17), anaplastic lymphoma kinase (ALK) (18), and neural cell adhesion molecule 1 (NCAM-1) (19), are being developed as targets for CAR T cell therapies. Only CAR T cell therapies against GD2, L1-CAM, and CD276 (Supplemental Table 1; supplemental material available online with this article; https://doi.org/10.1172/JCI155621DS1) have reached clinical trials and some, using CARs against GD2 and L1-CAM, have reported results (10–12, 14, 20, 21). Although these CARs in clinical trials have shown safety, they had limited efficacy and T cell persistence, with few patients achieving complete or partial responses (11, 12, 20, 21).

Besides GD2 and L1-CAM, recent studies implicate GPC2 and CD276 as promising cell-surface targets for immunotherapy (6, 15, 16, 22, 23). Both GPC2 and CD276 are significantly overexpressed in multiple pediatric cancers including NB, with low or undetectable expression in normal tissues (6, 15, 16, 22). GPC2 is critical for growth and differentiation of neurons in the developing nervous system (24, 25). CD276 is a checkpoint molecule involved in tumor immune evasion and metastasis (26). Augmented expression of CD276 is also observed in tumor blood vessels (22), and its overexpression correlates with poor prognosis in many cancers (27). In addition, the aforementioned CAR T cell clinical trials targeting CD276 in solid tumors including NB are currently under way. These characteristics and their location on the cell surface make them promising immunotherapeutic targets for NB.

Development of efficacious CAR T cell–based therapies requires the identification of optimized CARs, characterized by high cytotoxic activity, effective CAR T cell expansion, prolonged persistence, resistance to exhaustion in the presence of the target, and, ultimately, efficacy in shrinking or eradicating tumors. CAR constructs are developed using single-chain variable fragments (scFvs) typically derived from antibodies against the corresponding antigens. Therefore, properties of the scFv such as binding affinity, target-binding epitope/accessibility, and the propensity of the scFv to oligomerize affect CAR T cell activity (28–30). Despite several reports of effective targeting of GPC2 or CD276 by CARS in preclinical studies (6, 15, 31, 32), head-to-head comparisons have not been done to determine the performance of CARs. To address this, we developed a multimodal approach to identify optimized CARs among 14 CARs derived from previously reported or novel scFvs against GPC2 or CD276. First, we performed digital droplet PCR to measure T cell expansion, which was followed by pooled competitive optimization of CARs using cellular indexing of transcriptomes and epitopes by sequencing (CITE-Seq), termed P-COCC, to characterize CAR T cell activity profiles. This was combined with cytotoxicity assays to determine the tumor-killing ability of CAR T cells. Finally, we combined 2 identified optimal CARs targeting each tumor antigen to develop a bicistronic CAR (BiCisCAR) which overcame the heterogeneity of target expression for NB. We believe this multimodal approach can be utilized to screen for the optimal CAR constructs against candidate TAAs in a broad spectrum of solid tumors.

## Results

### GPC2 and CD276 are heterogeneously expressed in NB tumors and cell lines.

**Figure 1:**
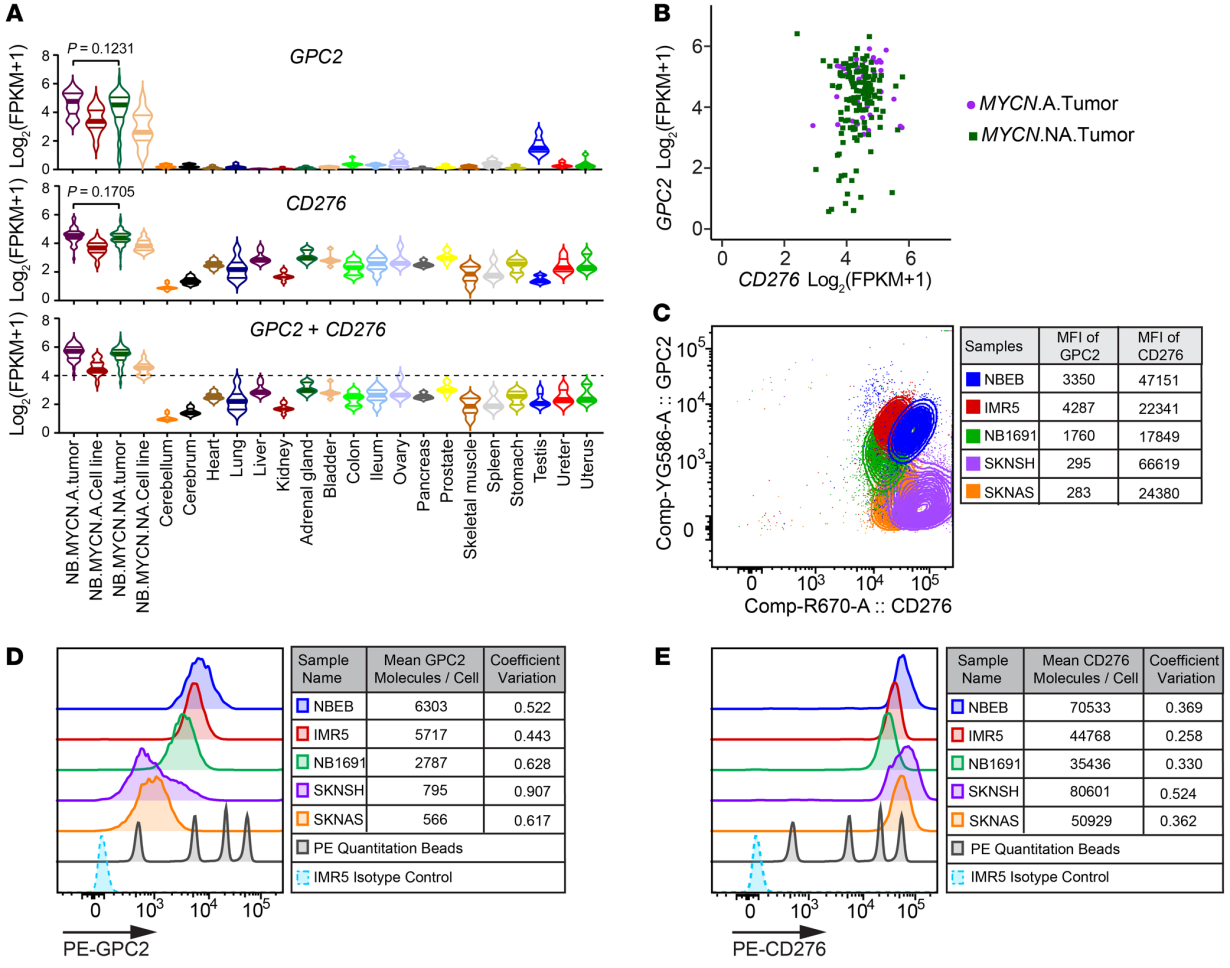
GPC2 and CD276 are highly and heterogeneously expressed on NB tumors and cell lines. (**A**) RNA-Seq showed high but heterogeneous expression of *GPC2* (top) and *CD276* (middle) in NB tumors and cell lines compared with expression in normal tissues. An ordinary 1-way ANOVA was used to calculate the *P* values. The bottom violin plot shows that combined expression of *GPC2* and *CD276* was high on approximately 95% of the NB samples. The horizontal line depicts the expression threshold cutoff compared with expression in normal tissues [log_2_(FPKM_[GPC2+CD276]_ +1) ≥4]. (**B**) Scatterplot of *GPC2* and *CD276* mRNA demonstrated a generally high level of expression for *CD276* but more variable expression of *GPC2* in NB tumors. (**C**–**E**) Flow cytometry was used to analyze GPC2 or CD276 expression on patient-derived NB cell lines by staining with anti–human GPC2 antibody (CT3 mAb) and anti–human/anti–mouse CD276 antibody (EPNCIR122, Abcam). Costaining of GPC2 and CD276 showed the heterogeneity of both targets on NB cells. The MFI for each target is quantified in the table (**C**). The expression of GPC2 or CD276 molecules on each NB cell was estimated with a phycoerythrin (PE) fluorescence quantitation kit (**D** and **E**). Tables show the quantification of protein expression measured by flow cytometric analysis from 3 independent experiments. The coefficient of variation is shown for the degree of heterogeneity of GPC2 and CD276 expression.

GPC2 and CD276 were recently identified as 2 promising targets for immunotherapy against NB (6, 15, 16, 32). However, heterogeneous expression of TAAs is a common phenomenon that leads to tumor immune escape and thus limits the efficacy of CAR T cell therapy against solid tumors (33, 34). Recent reports of tumor IHC have implied variable inter- and intratumoral expression of GPC2 and CD276 in NB (6, 17, 35). Using bulk RNA-Seq of 194 NB tumors and 40 NB cells lines, we confirmed that *GPC2* and *CD276* were highly expressed compared with expression levels in normal tissue but showed significant heterogeneity among samples (Figure 1, A and B). Furthermore, *GPC2* and *CD276* expression levels were independent of the tumor’s *MYCN* amplification status (Figure 1A). Combined expression analysis of GPC2 and CD276 showed high expression in approximately 95% of NB samples [log_2_(FPKM_[*GPC2*+*CD276*]_ +1) ≥4] (Figure 1A). However, we found that 32% of NB samples had low expression of *GPC2* [defined as log_2_(FPKM + 1) ≤4]. On the other hand, *CD276* was more consistently highly expressed in NB tumors, with only 18% of tumors expressing low levels (Figure 1B). To determine whether these observed patterns exist at the protein level, we measured the protein expression of GPC2 and CD276 on NB cell lines by flow cytometry (Figure 1C). First, we found that the protein expression levels of both targets was highly correlated with mRNA levels (*R^2^* = 0.9848 for GPC2 and *R^2^* = 0.5669 for CD276; Supplemental Figure 1, A and B). NBEB and IMR5 cells exhibited a high density of GPC2 and CD276 molecules on each cell, whereas SKNSH and SKNAS expressed GPC2 at a lower density, with a high CD276 density. NB1691 cells expressed both antigens at a medium density (Figure 1, D and E, and Supplemental Figure 1, C and D). Thus, our data suggested that combined CAR T cell therapies targeting both antigens simultaneously could be effective at addressing the heterogeneity of TAA expression seen in NBs.

### Expansion dynamics of 14 different CAR T cells targeting GPC2 or CD276.

Using a second-generation CAR design consisting of an extracellular scFv fused to the costimulatory domain 4-1BB coupled with CD3ζ, we constructed CARs targeting GPC2 or CD276 and then determined the expansion dynamics of the CARs in a pooled approach (Supplemental Figure 2, A and B). For CARs targeting GPC2, we used a total of 8 binders including 6 previously reported human VH single domains (LH1, LH2, LH3, LH4, LH6, and LH7) (15), 1 recently reported scFv CT3 derived from a mouse mAb (16), and 1 previously unreported scFv G27 obtained from a fully human antibody library (Supplemental Figure 2A). Six CD276 binders were constructed into CARs and tested, including (a) 2 m276 (VH-VL, VL-VH orientation) derived from a fully human antibody library (22); (b) 2 humanized scFv MGA271 derived from MacroGenics binder (VH-VL, VL-VH) (36); and (c) 2 humanized affinity-matured mAb 8H9 (VH-VL, VL-VH) (37) (Supplemental Figure 2B). The transduction efficiency, measured by GFP positivity, ranged from 22.6% to 61.5% for anti-GPC2 CAR T cells, and it ranged from 11.6% to 34.3% for anti-CD276 CAR T cells (Supplemental Figure 2C).

**Figure 2:**
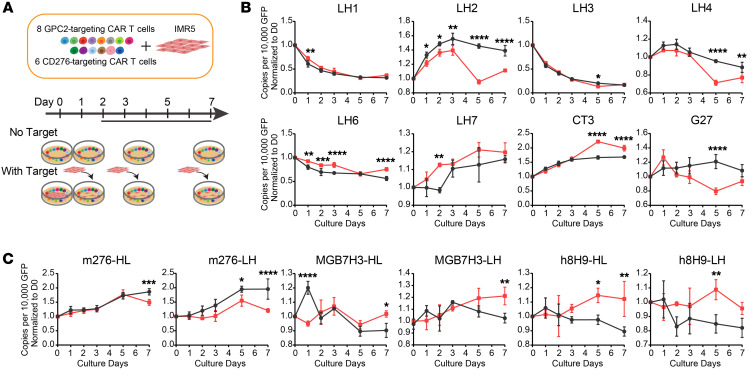
Measurement of the expansion of 14 distinct CAR T cells in a competition assay using ddPCR. (**A**) Experimental schema of a competition assay using a pool of 14 CAR T cells cocultured with or without tumor cells to monitor each CAR T cell expansion ability over time. (**B** and **C**) CT3 CARs showed maximal expansion among CARs targeting GPC2 when cocultured with targets (red squares), and 3 anti-CD276 CARs (MGB7H3-LH, h8H9-HL, and h8H9-LH) showed higher expansion levels than did their no-target controls (black circles). Copy numbers of each CAR in the pool of 14 CAR T cells over time were measured by ddPCR using specific primers against the scFv region of CAR to quantify each CAR accurately (*n* = 3, error bars indicate the SD). Each CAR copy per 10,000 GFP was then normalized to the value on day 0 (D0). *P < 0.05, **P < 0.01, ***P < 0.001, and ****P < 0.0001, by 2-way ANOVA with Sidak’s multiple-comparison test.

Robust expansion in the presence of its target is one of the characteristics of functional CAR T cells (38). We designed a competition assay pooling all 14 CAR T cells (8 anti-GPC2 and 6 anti-CD276) to determine the expansion capacity for each CAR (Figure 2A). Droplet digital PCR (ddPCR) was applied to precisely quantify the copy number of CAR constructs using specific PCR primers (Supplemental Table 2). CT3 CAR T cells showed the most significant expansion after stimulation with target IMR5 cells on days 5 and 7 (2.2-fold and 2-fold, respectively) when compared with their no-target controls (Figure 2B). Among the 6 anti-CD276 CARs, MGB7H3-LH, h8H9-HL, and h8H9-LH CAR T cells showed increased expansion in the presence of target compared with their no-target controls on day 5 or day 7 (Figure 2C). However, the fold increase was not as elevated as the anti-GPC2 CAR CT3. Thus, CT3 was selected as the top binder to the target GPC2, and all 3 candidate CARs targeting CD276 (MGB7H3-LH, h8H9-HL, and h8H9-LH) with improved expansion were selected for further characterization.

### P-COCC revealed individual CAR T cell activity signatures.

**Figure 3:**
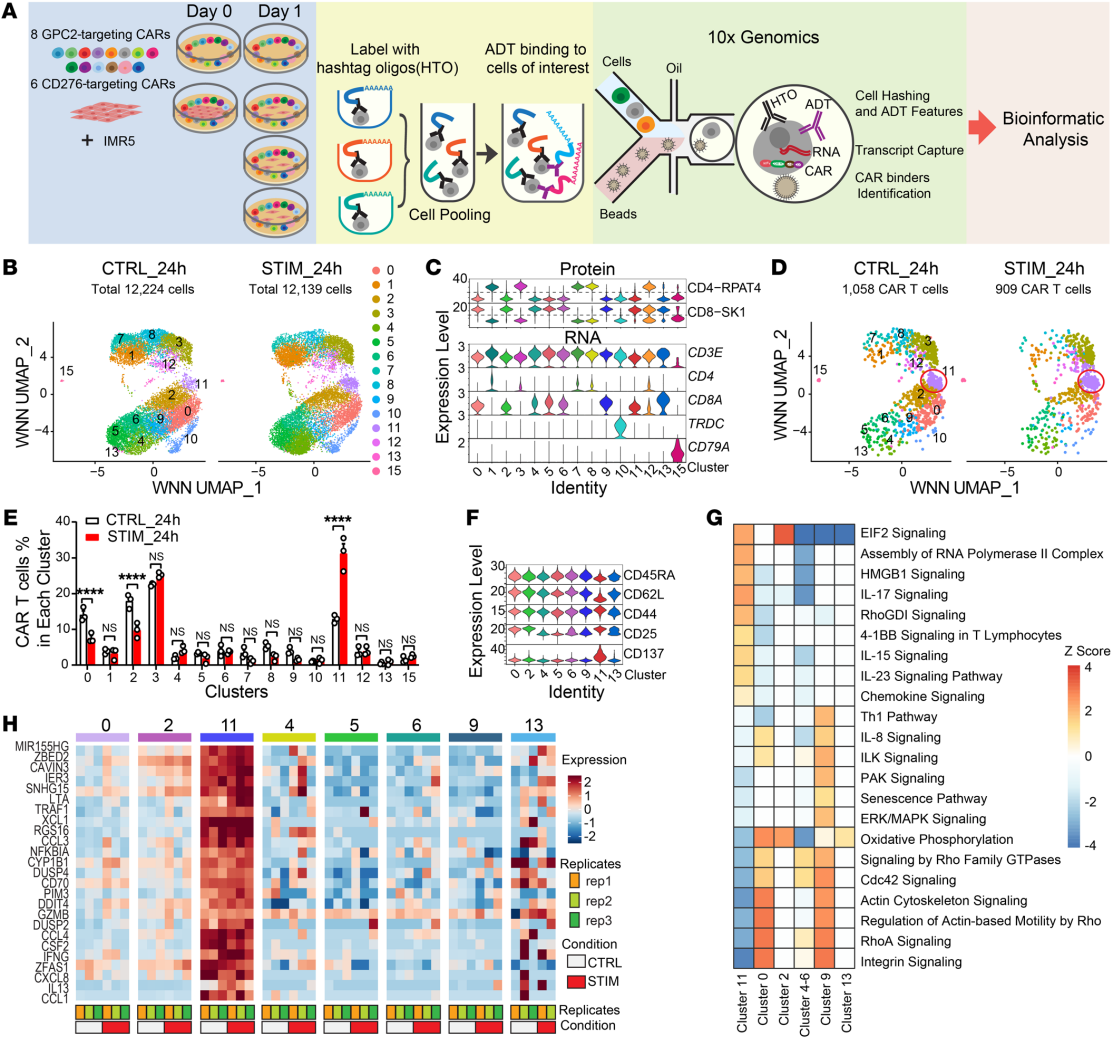
Multimodal single-cell profiling reveals significantly more T cells in cluster 11 of activated CD8^+^ effector T cells after stimulation. (**A**) Workflow of CITE-Seq for simultaneous protein and transcript analysis, combined with identification of 14 different anti-GPC2 or anti-CD276 CARs in the T cell pool. ADT, antibody-derived tag; HTO, hashtag oligonucleotide. (**B**) UMAP visualization of 12,224 and 12,139 T cells derived from 24-hour-cultured CAR T cells only (CTRL_24h) and CAR T cells cocultured with targets (STIM_24h), respectively, revealed 15 clusters with different transcriptome and protein profiles. (**C**) Violin plots of single-cell protein expression of the canonical CD4^+^ and CD8^+^ T cell markers among 15 clusters (upper panel) and RNA expression of canonical T cell and B cell genes (lower panel). Expression values on the upper panel represent denoised and scaled by background (DSB) normalized expression levels for each protein, whereas expression values on the lower panel are log scale–normalized RNA expression levels of genes. (**D**) WNN UMAP visualization of 1058 detected CAR T cells for CTRL_24h and 909 CAR T cells in STIM_24h samples. (**E**) The percentages of identified CAR T cell in each cluster from CTRL_24h and STIM_24h samples. Data points represent 3 replicates of each sample. ****P < 0.0001, by 2-way ANOVA with Šidák’s multiple-comparison test. (**F**) Violin plots of DSB-normalized surface protein markers show higher CD8^+^ T cell activation in cluster 11 compared with the other clusters. (**G**) Canonical pathways identified in each CD8 cluster by IPA. Colored scale bar reflects the predicted activation level (*z* <0, inhibited; *z* >0, activated; *z* ≥2 or ≤−2 can be considered significant). (**H**) Heatmap of the average expression of the top 25 cluster 11 genes in CAR T cells using scRNA-Seq data shows an activated CD8^+^ T cell phenotype consistent with protein markers. The colored scale bar represents *z* score values for gene expression.

To identify and characterize the activity of individual CAR T cells in the competition assay, we designed a pooled multimodal single-cell profiling approach termed P-COCC. Our assay simultaneously identified CAR T cells by sequencing the binding domain of CAR constructs and determined T cell functionality through CITE-Seq (39) using 28 protein markers (Supplemental Table 3 and Figure 3A).

To distinguish the identities of CAR T cells in the pool, we sequenced the single-cell barcoded library using PacBio, MiSeq, and PCR-based NextSeq, which applied similar TCR-VDJ sequencing methods. We detected 1778 CAR T cells within 4506 high-quality single cells without coculturing with tumor cells (CTRL_0h) (Supplemental Figure 3A). PacBio sequencing identified 1646 CAR T cells, while 791 were identified by MiSeq and 959 by PCR-based NextSeq, and one-third of them were detected by all 3 methods on day 0 (Supplemental Figure 3A). The frequency of identified CAR T cells in pooled T cells was 39.5%, which was consistent with the 33% CAR expression efficiency measured by GFP^+^ cells (Supplemental Figure 3B). Our data showed that we were able to determine the number and percentage of the CAR constructs on day 0 (Supplemental Figure 3, C and D). We also analyzed the phenotypic status of each of the 14 different CAR T cells identified through both surface protein antibodies, such as naive or memory T cell markers CD62L (*SELL*), CCR7, and CD27, and activated T cells markers CD44 and CD137 (*TNFRSF9*) combined with single-cell transcriptome expression. We found that the majority of CAR T cells were naive/resting T cells on day 0, as determined by CD62L positivity and CD137 negativity (Supplemental Figure 3, E and F).

We next compared CAR T cells stimulated with (STIM_24h) and without (CTRL_24h) NB IMR5 cells at the 24-hour time point. The experiment was performed using biological triplicates for each condition that were labeled by cell hashtags for identification. Using surface protein antibodies and single-cell RNA (scRNA) expression, we identified 12,224 and 12,139 T cells for stimulation and control experiments, respectively. CD8^+^ and CD4^+^ T cells were not clearly distinguishable from each other using transcriptome data alone (Supplemental Figure 4, A–C). Using weighted nearest-neighbor (WNN) analysis (see Methods) of integrated protein and transcriptome data, we identified 15 distinct clusters (Figure 3B and Supplemental Figure 4D). This multimodal integration analysis allowed us to clearly separate CD8^+^ T cells (including clusters 0, 2, 4, 5, 6, 9, 11, and 13) and CD4^+^ T cells (clusters 1, 3, 7, 8, and 12) (Supplemental Figure 4, E and F), and this was consistent with the expression of *CD4* and *CD8A* at the mRNA level (Figure 3C). In addition, we also determined γδ T cells as cluster 10 with high expression of *CD3E* and *TRDC,* and B cells as cluster 15 with *CD3E* negative and *CD79A* positive expression (Figure 3C). These results were consistent across biological triplicates, which gave us confidence in our method (Supplemental Figure 4G). Moreover, an increased number of protein molecules compared with RNA molecules led to a more robust detection of protein features at the single-cell level (Supplemental Figure 4H). Thus, integrative multimodal analysis of CITE-Seq data improved our ability to identify T cells and increased the robustness of the scRNA-Seq data.

We detected a total of 1058 CAR T cells in the CTRL_24h samples and 909 in the STIM_24h samples (Figure 3D). Among CAR T cells in all 15 clusters, only cells in cluster 11 (CD8^+^) significantly expanded after stimulation, from 13% to 31% (Figure 3E). Surface protein data analysis of CITE-Seq showed that CAR T cells in cluster 11 expressed higher levels of CD8A, CD25, CD44, and CD137, but had decreased expression of the naive markers CD45RA and CD62L, indicating that this cluster was composed of activated T cells (Figure 3F). Using differentially expressed genes (DEGs) to run Ingenuity Pathway Analysis (IPA), we identified the upregulation of HMGB1, RhoGDI, and 4-1BB signaling, which was elicited by T cell activation (Figure 3G). Cluster 11 showed transcriptomic evidence of lower oxidative phosphorylation (OXPHOS) compared with the other CD8 clusters, suggesting that these activated cells may have an altered metabolic profile (40) (Figure 3G). Indeed, measurement of oxygen consumption and glycolysis using Seahorse technology showed consistent metabolic differences with a skewing toward glycolysis rather than OXPHOS, using CT3 and LH2 as representative CAR T cells (Supplemental Figure 5, A–G). The top 25 DEGs in cluster 11 relative to other CD8^+^ CAR T cell clusters (fold change >2, Supplemental Table 5) included activation marker genes (*IFNG*, *MIR155HG*, *ZBED2*, and *GZMB*) and multiple inflammatory chemokine or cytokine genes (*IL13*, *CCL3*, *CCL4*, and *CXCL8*), representing CAR T cell polyfunctionality (Figure 3H). These results suggested that CAR T cells in cluster 11 were activated CD8^+^ effector T cells that markedly increased in frequency after stimulation by IMR5 cells for 24 hours.

**Figure 4:**
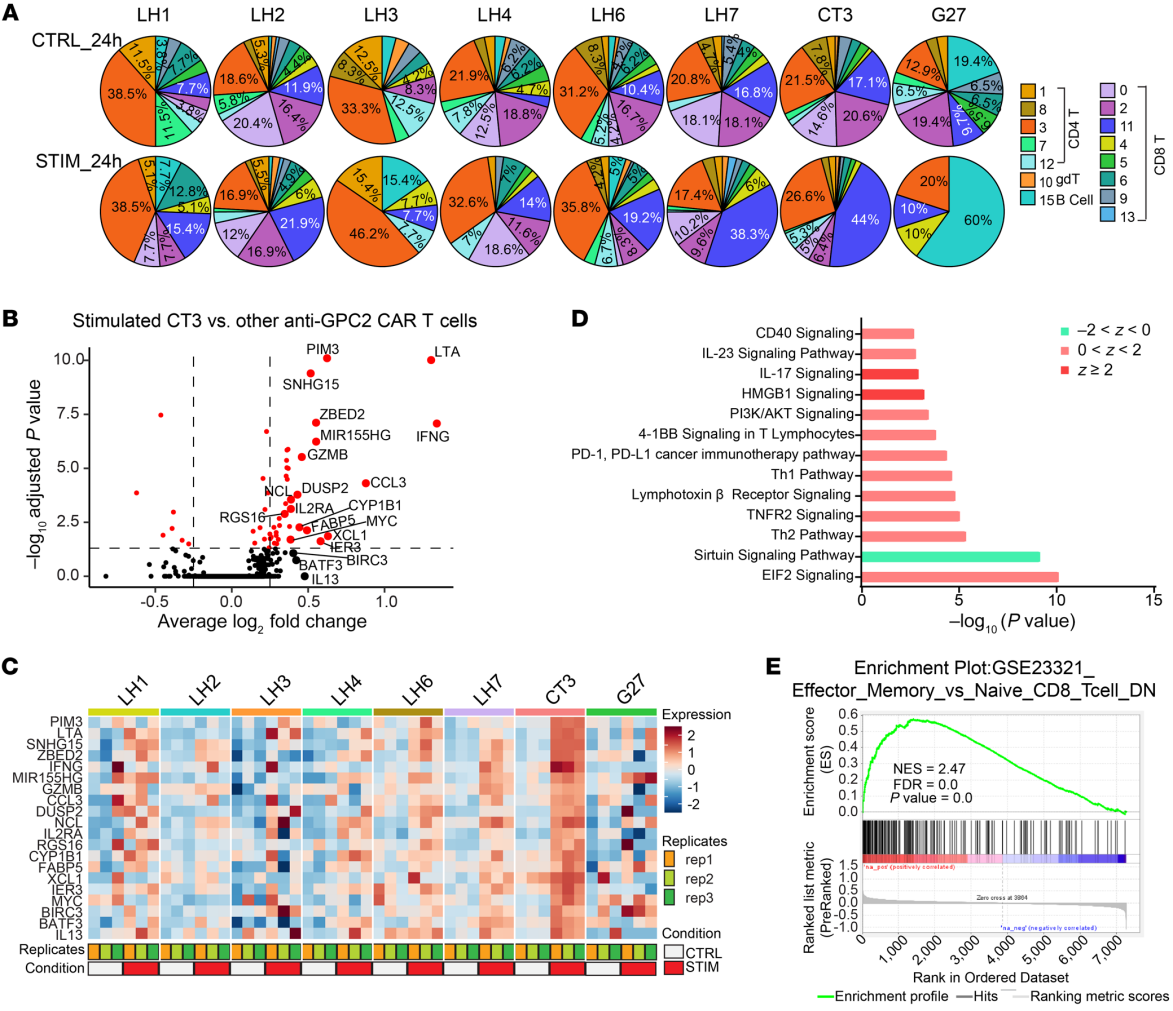
P-COCC identifies the optimal anti-GPC2 CAR CT3 with higher activation and antitumor polyfunctionality signatures. (**A**) Pie charts show the proportions of 15 clusters within 8 types of anti-GPC2 CAR T cells. Only identified CAR T cells were taken into count. Clusters are distinguished by colors in the color key. (**B**) Volcano plot of DEGs between the CT3 CAR and 7 other anti-GPC2 CARs 24 hours after coculturing with IMR5 cells. Genes with an adjusted *P* value of less than 0.05 are shown in red. The top 20 genes, ranked by average log_2_ fold change according to a nonparametric Wilcoxon rank-sum test, are labeled on the plot. (**C**) Heatmap shows averaged expression of the top 20 genes in 8 different anti-GPC2 CARs within a CAR T cell pool cocultured with IMR5 cells (STIM_24h) or without targets (CTRL_24h) for 24 hours. (**D**) Canonical signaling pathways regulated by the DEGs identified in **B**. A positive or negative *z* score value indicates that a pathway is predicted to be increased or decreased, respectively, in stimulated CT3 CARs relative to other 7 GPC2-targeting CARs. (**E**) GSEA of effector memory T cell genes in a ranked fold change list of DEGs between CT3 CARs and the other 7 anti-GPC2 CAR T cells, 24 hours after stimulation with IMR5 cells. NES, normalized enrichment score.

To assess which CAR T cells had the highest activity, we next measured the change in CD8^+^ effector T cells (cluster 11) for all 14 CAR T cell types. Among 8 anti-GPC2 CARs, the frequency of cluster 11 increased the most in CT3 CAR T cells after a 24-hour stimulation with IMR5 cells, from 17% to 44% (Figure 4A and Supplemental Figure 6A). Compared with 7 other anti-GPC2 CAR T cells, stimulated CT3 CAR T cells significantly upregulated the expression of genes involved in antitumor activity (*IFNG*, *CCL3*, and *GZMB*) and proliferation ability (*MIR155HG*, *MYC*, *ZBED2*) (Figure 4, B and C, and Supplemental Table 6). IPA showed CAR T cell activation (HMGB1, PI3K/AKT, and CD40 signaling) with the capability of producing multiple cytokines through IL-17, IL-23, Th1, and Th2 pathways (Figure 4D). Gene set enrichment analysis (GSEA) of these DEGs in CT3 CAR T cells showed significant enrichment of effector memory T cell profiles (Figure 4E). Therefore, these data suggested that CT3 CAR T cells had the greatest capacity for activation, proliferation, and antitumor activity among anti-GPC2 CAR T cells.

Likewise, we were able to detect differences among anti-CD276 CAR T cells (Supplemental Figure 6, B–F). Among the 6 anti-CD276 CARs, MGB7H3-LH CAR demonstrated higher expression of chemokine genes (*CCL3*, *CCL4, CCL3L1*, and *CCL4L2*) after stimulation by IMR5 cells compared with the other 5 constructs (Supplemental Figure 6, B–F, and Supplemental Table 7). Overall, our approach using P-COCC revealed CT3 and MGB7H3-LH as the top CAR T cells with activated effector T cell signatures and robust polyfunctionality among anti-GPC2 and anti-CD276 CARs, respectively.

### Cytotoxicity assays confirm CT3 and MGB7H3-LH as the most effective CAR constructs targeting GPC2 and CD276, respectively.

**Figure 5:**
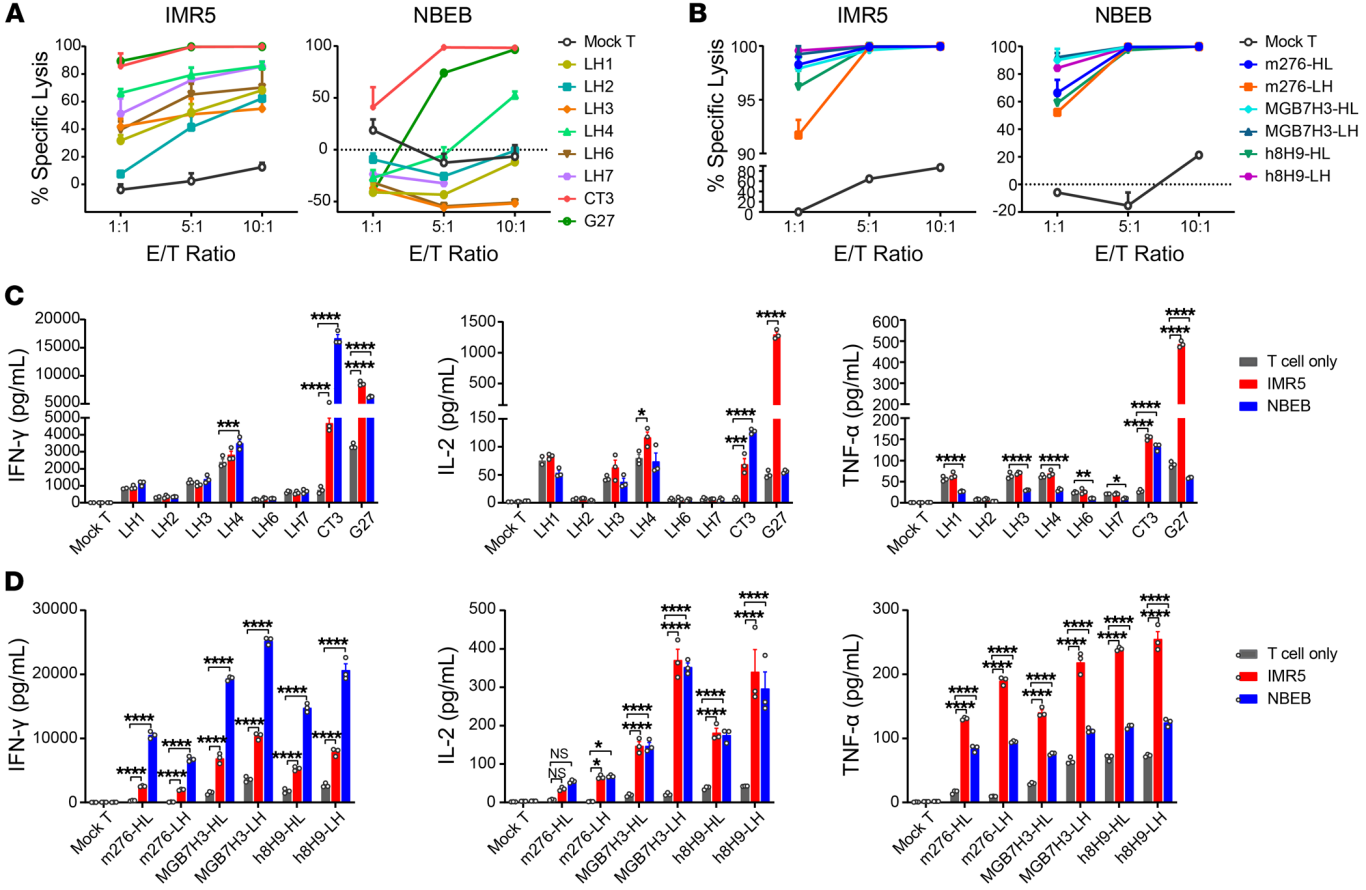
Cytotoxicity assays of 14 CARs validate CT3 as the most effective GPC2-targeting CAR and MGB7H3-LH as the most effective CD276-targeting CAR. (**A**) Eight anti-GPC2 CAR T cells were separately cocultured for 20 hours in vitro with IMR5 (*MYCN*-A) or NBEB (*MYCN*-NA) cells engineered to express luciferase. The specific lysis percentages of NB cells were measured by luciferase assay. (**B**) Cytotoxicity assays of 6 individual CD276-targeting CAR T cells cocultured in vitro with IMR5 (*MYCN*-A) or NBEB (*MYCN*-NA) cells. (**C**) IFN-γ, IL-2, and TNF-α production after 20 hours of coculturing the 8 anti-GPC2 CAR T cells and NB cells. (**D**) Levels of IFN-γ, IL-2, and TNF-α released by the 6 CD276-targeting CAR T cells following a 20-hour coculture with IMR5 or NBEB cells. Data are shown as individual values and the mean ± SEM; *n* = 3 independent coculture with CAR T cells. *P < 0.05, **P < 0.01, ***P < 0.001, and ****P < 0.0001, by 2-way ANOVA with Dunnett’s multiple-comparison test (**C** and **D**).

After characterizing the expansion ability and expression profiles of these CAR T cells, we next examined which CAR T cell was most effective at killing NB cells. We measured the efficacy of the 14 CAR T cells against luciferase-expressing NB tumor cells through a bioluminescence-based cytotoxicity assay in vitro (Figure 5, A and B). CT3 CAR T cells mediated the most effective killing of NBEB cells expression high levels of GPC2 at an effector/target (E/T) ratio of 1:1 and was accompanied by significant production of antitumor effector cytokines, including IFN-γ, IL-2, and TNF-α (Figure 5, A and C). Several of the other GPC2-targeting CAR constructs also showed tumor-killing ability. However, this activity was likely nonspecific, since they induced high expression of IFN-γ, TNF-α, and IL-2 with and without target NB cell stimulation (Figure 5, A and C). Despite tumor cell–killing ability and cytokine production comparable to those of CT3 CAR T cells, G27 CAR T cells showed high tonic signaling in the absence of targets (Figure 5, A and C). Combined with the lack of expansion and activity signatures, the G27 CAR was therefore eliminated as an optimally functional CAR. Thus, GPC2-specific NB killing of CT3 CAR T cells was consistent with the highest expansion capacity and activity profile, as determined by P-COCC analysis, which validated CT3 CAR as the most effective anti-GPC2 CAR.

For anti-CD276 CARs, all 6 candidate CAR T cells showed highly specific killing activity against NB cells (Figure 5B). Among them, MGB7H3-LH CAR T cells showed the highest killing activity against NB cells concurrently with the most IFN-γ and IL-2 production (Figure 5D). Thus, our P-COCC analysis was consistent with both the cytotoxic and cytokine assays demonstrating that MGB7H3-LH was the most effective anti-CD276 CAR construct.

### BiCisCAR T cells show potent activity in killing GPC2- and CD276-coexpressing NB cells in vitro.

**Figure 6:**
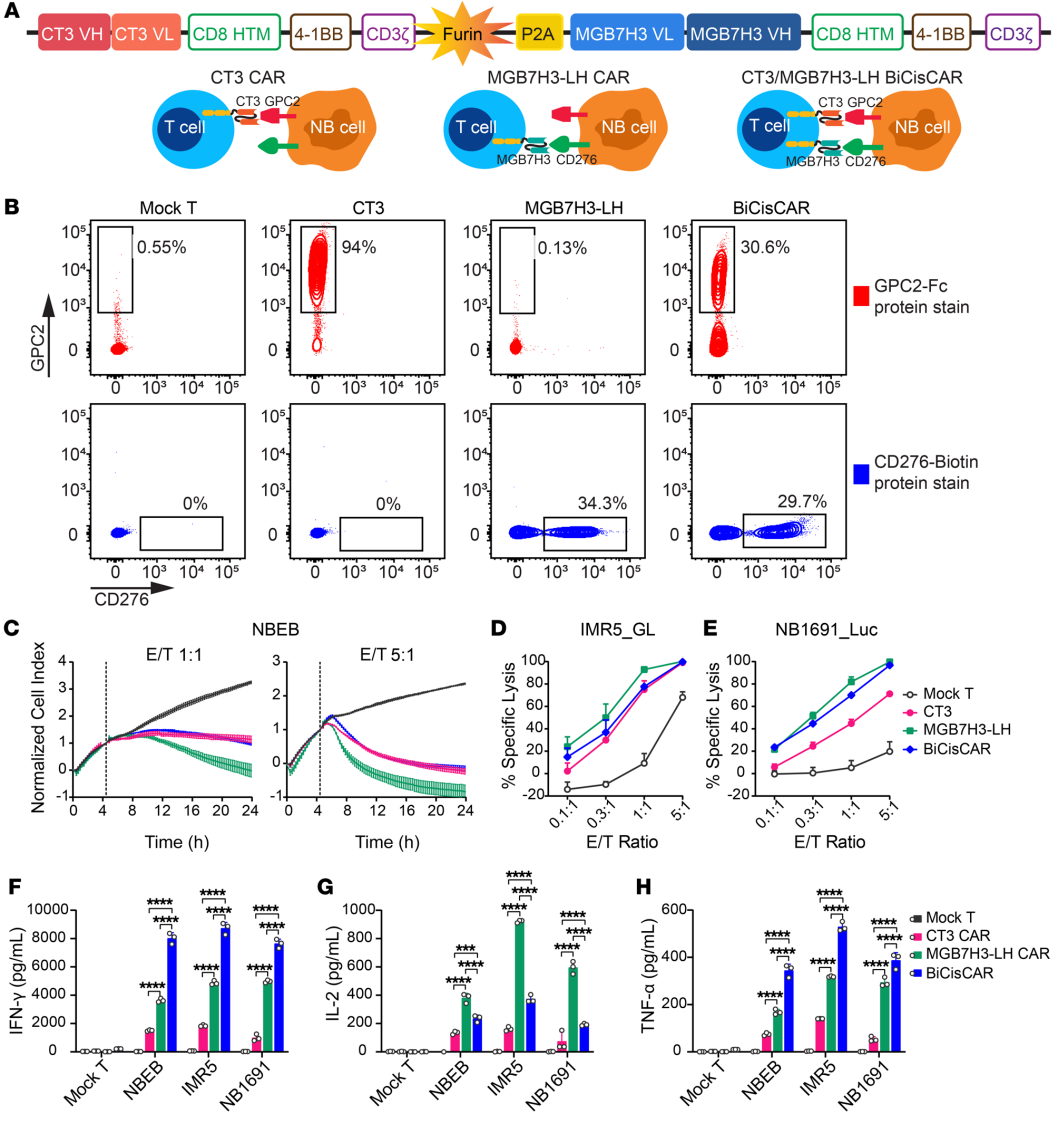
BiCisCAR T cells targeting both GPC2 and CD276 show potent NB cell–killing activity in vitro. (**A**) Schematic of designed BiCisCAR structure targeting either GPC2 or CD276 on NB cells. HTM, hinge and transmembrane domain; ICD, intracellular domain. (**B**) Representative flow cytometric plots show CAR T cells binding with Fc-GPC2 chimeric protein (top, red) or biotinylated human CD276 protein (bottom, blue) separately. (**C**) Cytotoxicity of 3 CAR T cells after coculturing with NBEB cells at the indicated E/T ratio in an xCELLigence real-time cell analysis (RTCA) assay. (**D** and **E**) Three CAR T cells were individually cocultured for 20 hours with luciferase-expressing IMR5 or NB1691 cells at the indicated E/T ratio, and the specific lysis percentages of tumor cells were detected by luciferase assay. (**F**–**H**) IFN-γ (**F**), IL-2 (**G**), and TNF-α (**H**) released by 3 types of CAR T cells following a 20-hour coculture with NBEB, IMR5, or NB1691 cells. Data are shown as individual values and the mean ± SD; *n* = 3 independent coculture with CAR T cells. ***P < 0.001 and ****P < 0.0001, by 2-way ANOVA with Tukey’s multiple-comparison test (**F**–**H**).

Given the heterogeneity of GPC2 and CD276 on NB cells (6, 35), targeting only 1 antigen may be ineffective at eliminating the tumor, leading to immune evasion (41, 42). Having identified CT3 and MGB7H3-LH as the top 2 functional CAR constructs targeting GPC2 and CD276, respectively, we next developed a BiCisCAR that had these 2 complete CAR constructs connected with a cleavable T2A sequence and used 4-1BB costimulatory domains for both binders, allowing coexpression of both constructs in the same T cell (43) (Figure 6A). Flow cytometric analysis demonstrated this construct’s ability to bind both GPC2 and CD276 proteins (Figure 6B). An in vitro cytolytic assay illustrated that this BiCisCAR had a killing ability comparable to that of NB cells with a high density of GPC2 and CD276 (NBEB and IMR5) and medium levels of antigen density (NB1691) (Figure 6, C–E). When compared with 2 single antigen–targeting CAR T cells, the BiCisCAR T cells released the highest level of the cytokines IFN-γ and TNF-α, 20 hours after coculturing with 3 NB cell lines (Figure 6, F–H). Thus, this BiCisCAR recognized both GPC2 and CD276 and showed potent killing activity against NB cells coexpressing both antigens.

### GPC2/CD276 BiCisCAR exhibits superior antitumor activity to tumor cells expressing either GPC2 or CD276.

**Figure 7:**
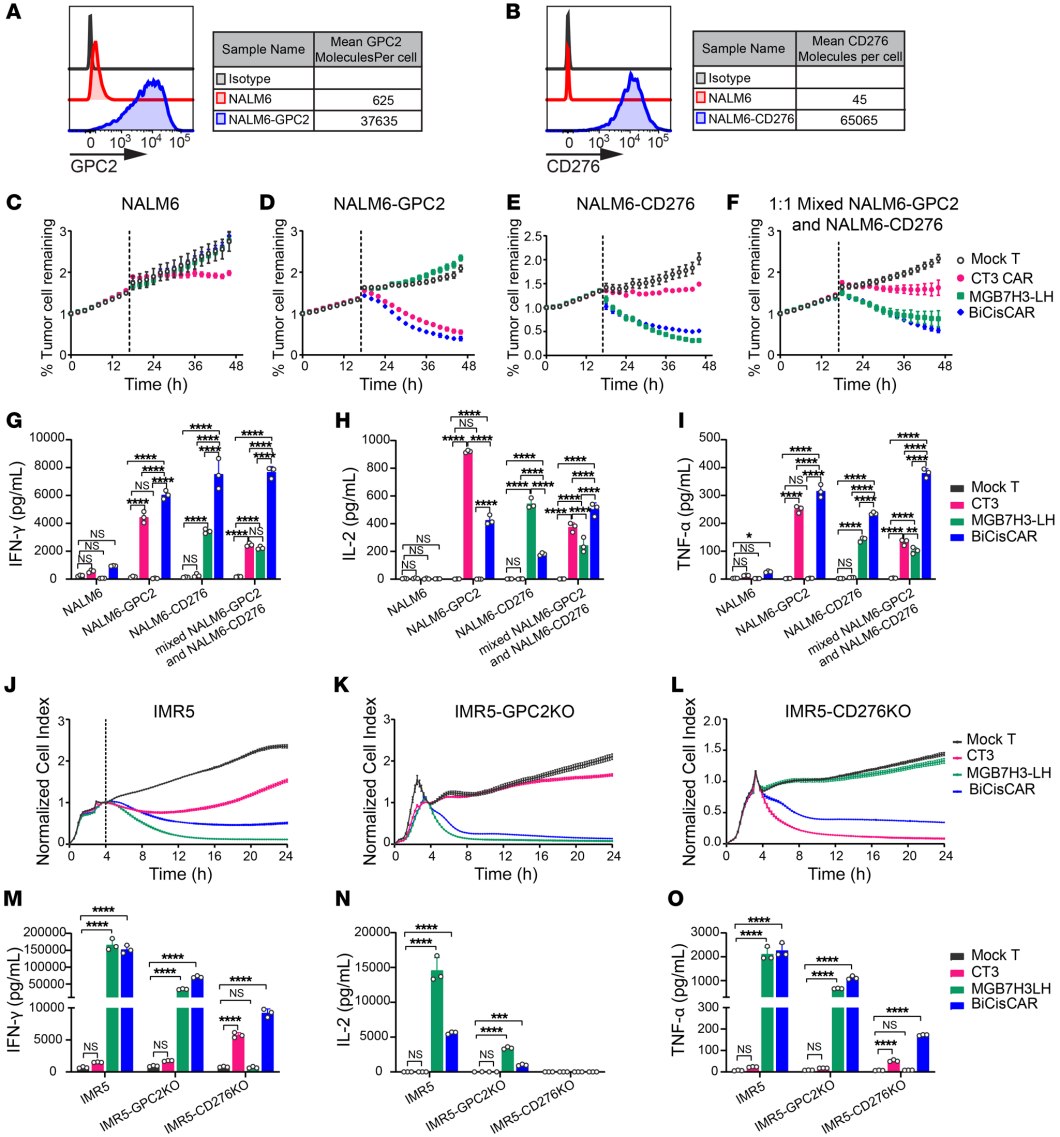
GPC2/CD276 BiCisCAR shows superior antitumor activity when targeting either NALM6 cells expressing GPC2 or CD276 or GPC2-KO or CD276-KO NB cells. (**A** and **B**) Representative flow cytometric plots of the levels of GPC2 or CD276 expression on NALM6 leukemia cells or on NALM6 cells stably transduced with GPC2 or CD276. GPC2 or CD276 molecules expressed per NALM6 cell were quantified using a PE Quantitation kit. (**C**–**F**) GFP^+^ NALM6 cells, NALM6-GPC2 clones, NALM6-CD276 clones, or 1:1 mixed NALM6-GPC2 and NALM6-CD276 cells cocultured with single antigen–targeting CARs or GPC2/CD276 BiCisCARs separately. An IncuCyte assay was performed to measure tumor cell–killing dynamics over 48 hours. Representative data from 3 experiments are shown. (**G–I**) Summary of IFN-γ (**G**), IL-2 (**H**), and TNF-α (**I**) released by mock, CT3 CAR, MGB7H3-LH CAR, and GPC2/CD276 BiCisCAR T cells in the cultured supernatant after 20 hours of coculturing with the indicated cell lines (*n* = 3). Error bars indicate the SD. (**J**–**L**) Activities of single CARs or GPC2/CD276 BiCisCARs were evaluated in vitro with ACEA killing assays against IMR5 cells and IMR5 cells with CRISPR/Cas9 KO of GPC2 or CD276. (**M**–**O**) Cytokine production by CT3 CAR, MGB7H3-LH CAR, and BiCisCAR T cells after coculturing with IMR5, GPC2-KO IMR5, or CD276-KO IMR5 cell lines. Data are shown as individual values and the mean ± SD; *n* = 3 independent cocultures with CAR T cells. *P < 0.05, **P < 0.01, ***P < 0.001, and ****P < 0.0001, by 2-way ANOVA with Tukey’s multiple-comparison test (**G**–**I** and **M**–**O**).

To further validate that the BiCisCAR T cells can eliminate cells expressing either GPC2 or CD276, NALM6 cells, a line of B cells that are GPC2^lo^CD276^–^, were used to overexpress GPC2 or CD276 separately and stably (Figure 7, A and B). Single GPC2–targeting CAR CT3 and BiCisCAR, but not CD276 CAR MGB7H3-LH, were able to elicit an effective cytotoxic response to GPC2-overexpressing NALM6 cells in vitro (Figure 7, C, D, and G–I, and Supplemental Figure 7A). Similarly, single CD276–targeting CAR and BiCisCAR, but not GPC2-targeting CAR, showed efficient killing activity against CD276-overexpressing NALM6 cells (Figure 7, E and G–I, and Supplemental Figure 7B). Interestingly, these BiCisCAR T cells exhibited superior antitumor activity and produced significantly higher IFN-γ and TNF-α in the presence of both GPC2-NALM6 and CD276-NALM6 cells compared with 2 single antigen–targeting CARs (Figure 7, F and G–I, and Supplemental Figure 7C).

To further examine the ability of the BiCisCAR to overcome the heterogenous expression of GPC2 and CD276 on NB, we next used CRISPR/Cas9 to create GPC2- or CD276-null mutations in 2 NB cell lines (IMR5 and IMR32). When GPC2 was deleted, anti-GPC2 CAR CT3 lost its killing ability and produced levels of cytotoxic cytokines comparable to those of mock T cells (untransduced T cells) (Figure 7, K and M–O, and Supplemental Figure 7, E and H–J). Similarly, anti-CD276 CAR MGB7H3-LH CAR T cells were not able to kill CD276-KO IMR5 or IMR32 cells or release specific antitumor cytokines (Figure 7, L–O, and Supplemental Figure 7, F and H–J). Alternatively, GPC2/CD276 BiCisCAR T cells displayed effective killing activity against GPC2-KO or CD276-KO NB cells, except when both targets were knocked out in IMR32 cells (Supplemental Figure 7, G and H–J). We observed a modest reduction of killing ability of BiCisCAR T cells against IMR5 cells compared with anti-CD276 CAR T cells, which was attributed to a lower density of CD276 binders in CAR T cells transduced with BiCisCARs (Figure 7J and Supplemental Figure 8, D–F). Despite this, the BiCisCAR T cells showed superior specific antitumor activity in the absence of either GPC2 or CD276 antigens.

To determine whether GPC2 and CD276 binders on BiCisCARs are additive or synergistic, we performed flow cytometry to measure intracellular cytokine production after single antigen activation or dual activation with GPC2-KO IMR5 or CD276-KO IMR5 cells or dual activation with IMR5 cells. When both binders were activated by IMR5 cells, the percentage of cytokine-producing BiCisCAR T cells was approximately equal to the sum of those activated by GPC2-KO or CD276-KO IMR5 cells individually (Supplemental Figure 8, G–K). Therefore, these cytokine-release data demonstrated an additive relationship between 2 binders on BiCisCAR T cells.

### BiCisCARs exhibit efficacy comparable to that of anti-CD276 CAR T cells but are characterized by more central memory T cells.

We used NBEB cells with high coexpression of GPC2 and CD276 to test preclinical efficacy of this BiCisCAR in a s.c. xenograft model of NB (Supplemental Figure 9A). We found that anti-GPC2 CAR CT3 was able to partly suppress this solid tumor growth, but anti-CD276 CAR MGB7H3-LH and BiCisCAR showed comparable efficacy in shrinking and eliminating tumors (Supplemental Figure 9, B and C). Mice treated with anti-CD276 single CARs or BiCisCARs had a significant survival advantage compared with anti-GPC2 single CAR or mock T cell–treated mice (Supplemental Figure 9D).

**Figure 8:**
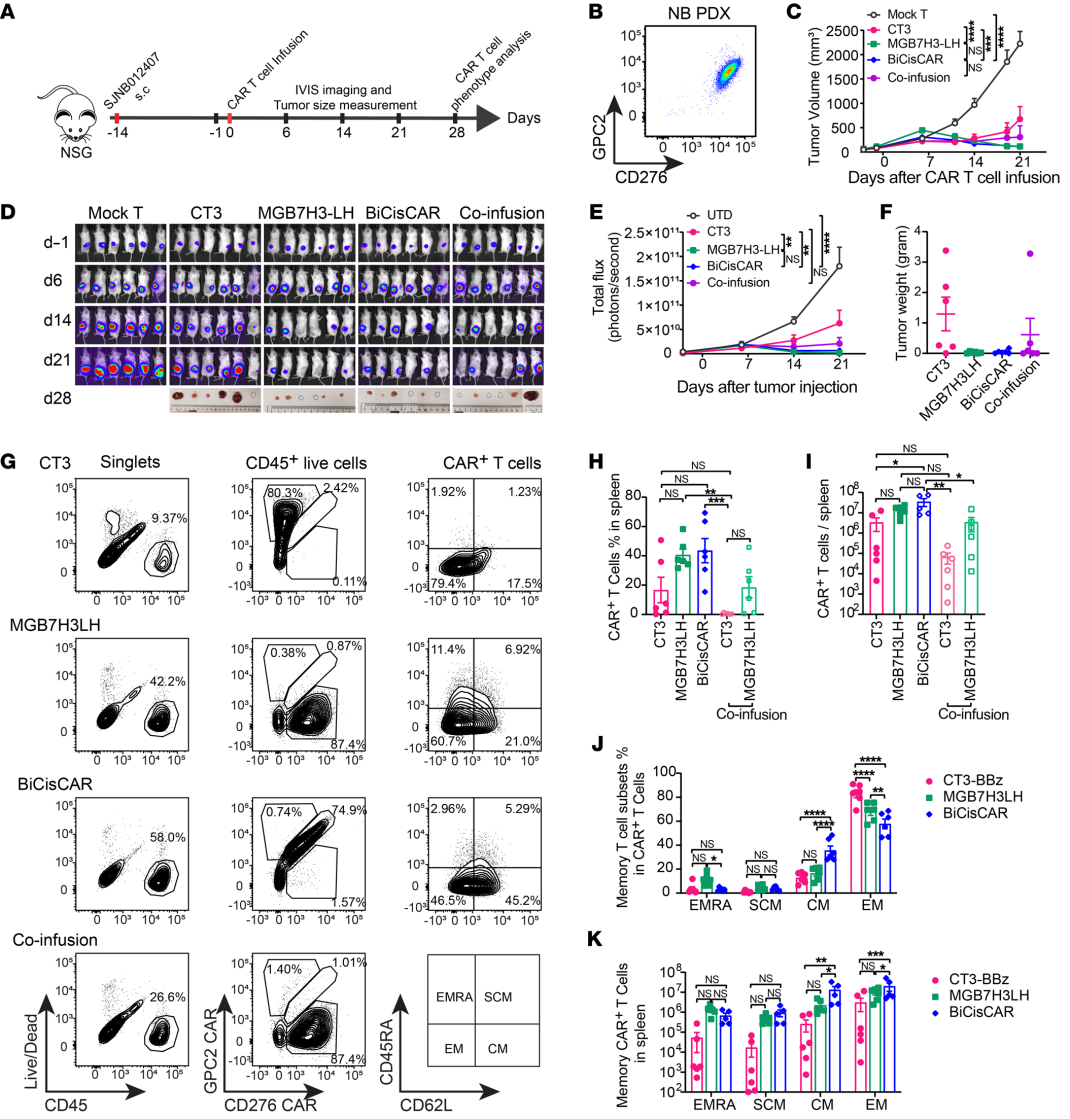
Single GPC2 or CD276–targeting CAR or BiCisCAR treatment of a s.c. NB PDX model. (**A**) Schema of a NB PDX model infused with CAR T cells on day 14 after tumor inoculation. (**B**) Representative flow cytometric plot shows high GPC2 and CD276 expression in SJNB012407 PDX cells dissociated from the xenograft tumor. (**C**) Tumor volumes following CAR T cell infusion. Data indicate the mean ± SEM of tumor volume (*n* = 6). ***P < 0.001 and ****P < 0.0001, by 2-way, RM ANOVA. (**D**) Representative bioluminescence images of SJNB012407 tumor growth. (**E**) Bioluminescence kinetics of NB PDX showed tumor progression after CAR T cell treatment using total flux values (photons per second). **P < 0.01 and ****P < 0.0001, by 2-way repeated-measures (RM) ANOVA. (**F**) Weight of each tumor from mice treated with CAR T cells for 28 days (data indicate the mean ± SEM). (**G**) Representative flow cytometric plots show the percentage of CAR^+^ T cells in splenocytes from mice 28 days after CAR T cell infusion and the percentage of CAR T cells in different memory states (right column, *n* = 6, data indicate the mean ± SEM). (**H** and **I**) Percentage of CAR T cells in splenic lymphocytes (**H**) and total counts of the indicated CAR T cells in whole spleens (**I**). Data are shown as the mean ± SEM (*n* = 6). *P < 0.05, **P < 0.01, and ***P < 0.001, by 1-way ANOVA with Tukey’s multiple-comparison test. (**J** and **K**) Percentage of CAR T cells in different memory cell states (**J**) and total counts of memory T cells in the whole spleen (**K**). Data indicate the mean ± SEM (*n* = 6). *P < 0.05, **P < 0.01, ***P < 0.001, and ****P < 0.0001, by 2-way ANOVA with Tukey’s multiple-comparison test. CM, central memory T cells; EM, effector memory T cells; EMRA, terminally differentiated effector memory cells reexpressing CD45RA; SCM, stem cell memory T cells.

To further determine BiCisCAR T cell efficacy, we used a GPC2- and CD276-coexpressing NB patient–derived xenograft (PDX) in a s.c. mouse model (Figure 8, A and B). We first examined T cell infiltration in tumors on day 11 after infusion of CAR T cells (Supplemental Figure 10A). Except for the mock T cells, all CAR T cells showed good tumor infiltration prior to tumor shrinkage (Supplemental Figure 10A). However, consistent with the NBEB model, anti-GPC2 CAR T cells showed the lowest efficacy among all CAR T cells (Figure 8, C–F). Although coinfusion of anti-GPC2 and anti-CD276 CAR T cells moderately suppressed or even eliminated tumors in 33.3% of the mice, anti-CD276 CARs and BiCisCARs still demonstrated better efficacy (Figure 8, C–F). To investigate the persistence of CAR T cells, we performed flow cytometry on CAR T cells isolated from mouse spleens. We found that there were lower percentages and counts of anti-GPC2 CAR T cells remaining in the spleens from mice treated with CT3 only or with coinfusion of CAR T cells, suggesting that anti-GPC2 CAR T cells did not persist in vivo. Moreover, GPC2- and CD276-targeting CAR T cells did not show a similar persistence in the coinfusion experiments compared with BiCisCAR T cells (Figure 8, G–I). Furthermore, BiCisCAR T cells displayed higher percentages and counts as central memory T cells on day 28 than did other single CARs (Figure 8, J and K, and Supplemental Figure 10B), suggesting long-term antitumor activity.

### BiCisCAR T cells exhibit superior efficacy in a metastatic heterogeneous model, with prolonged persistence and reduced exhaustion.

**Figure 9:**
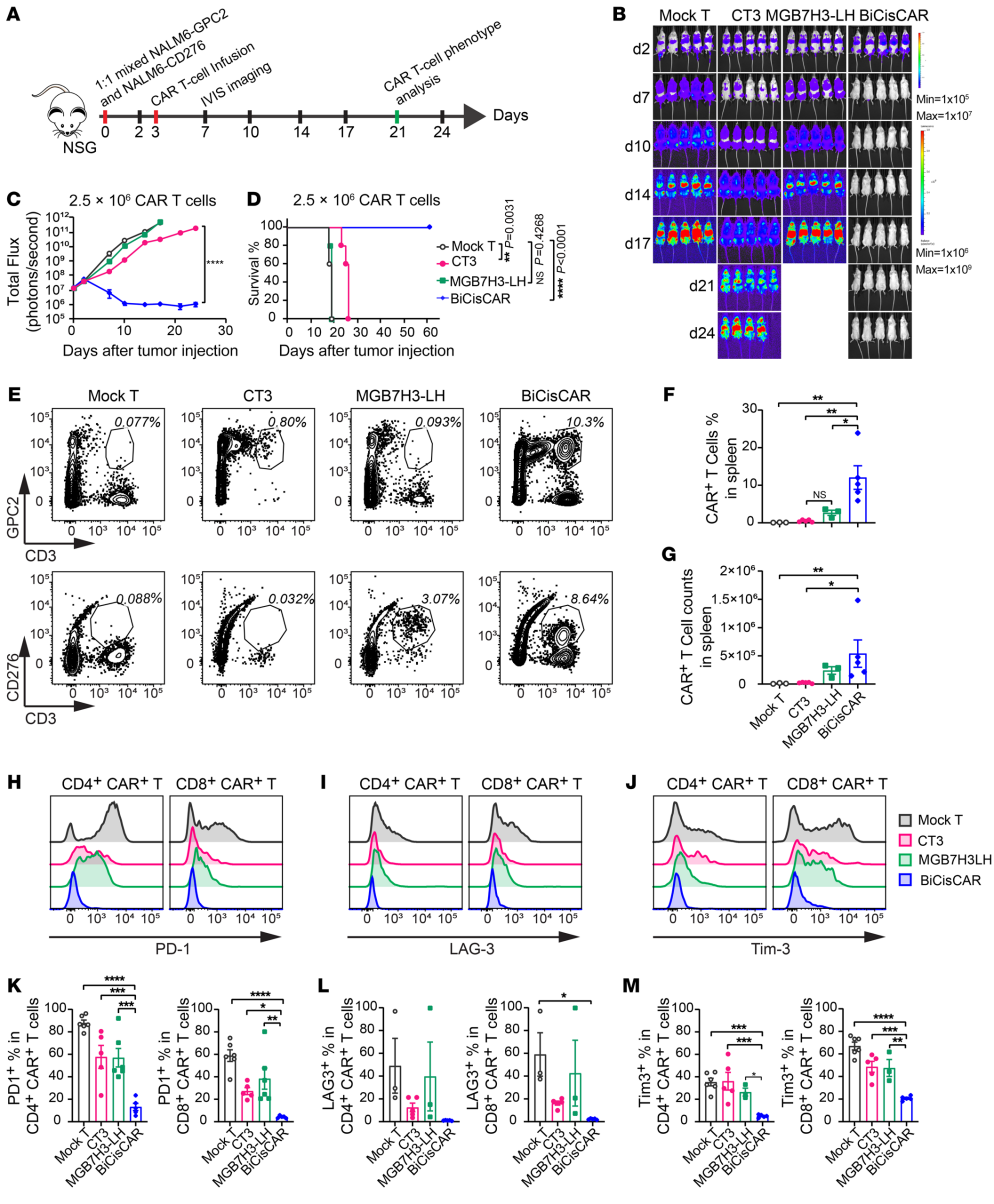
BiCisCARs show superior efficacy in eradicating tumor cells heterogeneously expressing GPC2 or CD276, improved T cell persistence, and reduced T cell exhaustion. (**A**) Schema of the metastatic tumor model with 1:1 mixed NALM6-GPC2 and NALM6-CD276 cells infused with 2.5 × 10^6^ or 5 × 10^6^ CAR T cells on day 3 after tumor inoculation. (**B** and **C**) Representative bioluminescence images (**B**) and bioluminescence kinetics (**C**) of NALM6 cell growth before (d2) and after (≥d7) infusion with 2.5 × 10^6^ CAR T cells. ****P < 0.0001, by 2-way RM ANOVA. (**D**) Kaplan-Meier survival analysis of mice treated with CAR T cells (*n* = 5 mice/group). **P < 0.01 and ****P < 0.0001, by log-rank test. (**E**) Representative flow cytometric plots of CAR T cell frequencies in spleens from the mice described above, 21 days after infusion with 5 × 10^6^ CAR T cells. (**F**) The percentages of CAR T cells in spleens from mice treated with 5 × 10^6^ CAR T cells were analyzed by flow cytometry (*n* = 5; data indicate the mean ± SEM). *P < 0.05 and **P < 0.01, by 1-way ANOVA with Dunnett’s multiple-comparison test. (**G**) Total numbers of the indicated CAR T cells in whole spleens from mice treated with 5 × 10^6^ CAR T cells. *P < 0.05 and **P < 0.01, by nonparametric Kruskal-Wallis test. (**H**–**J**) Representative flow cytometry illustrating PD-1 (**H**), LAG-3 (**I**), and Tim-3 (**J**) expression in CD4^+^ or CD8^+^ CAR T cells in spleens of mice from the NALM6 metastatic model, 21 days after infusion of 5 × 10^6^ CAR T cells (*n* = 5, mean ± SEM). (**K**–**M**) Percentages of PD-1 (**K**), LAG-3 (**L**), and Tim-3 (**M**) in CD4^+^ or CD8^+^ CAR T cells in mice 21 days after infusion with 5 × 10^6^ CAR T cells (*n* = 5, mean ± SEM). *P < 0.05, **P < 0.01, ***P < 0.001, and ****P < 0.0001, by 1-way ANOVA, Dunnett’s multiple-comparison test.

To model the heterogenous expression of GPC2 and CD276, which can result in tumor evasion in single-target CAR T cell therapies, we used a mixture of NALM6 cells stably expressing either GPC2 or CD276 and treated them with BiCisCARs or single antigen–targeting CARs (Figure 9A and Supplemental Figure 11A). As expected, single antigen–specific CAR T cells could not suppress tumor growth, although the GPC2 single CAR performed slightly better than did the CD276 single CAR because of the low expression levels of GPC2 in the CD276-overexpressing NALM6 cells (Figure 9B and Supplemental Figure 11, B and C). Importantly, in vivo imaging showed that both 2.5 × 10^6^ and 5 × 10^6^ doses of BiCisCAR T cells completely eradicated leukemia expressing GPC2 or CD276 and prevented the recurrence of leukemia (Figure 9, B and C, and Supplemental Figure 11, B and C). Flow cytometry showed that neither GPC2- nor CD276-expressing NALM6 cells were detectable in NSG mice treated with BiCisCAR T cells (Supplemental Figure 11D). These mice also had markedly prolonged survival (Figure 9D). These in vivo experiments illustrated the improved antitumor capacity of the BiCisCAR T cells compared with the single-target CAR T cells.

Two common challenges in CAR T cell therapy are limited T cell persistence and T cell exhaustion. Therefore, we next examined whether BiCisCAR T cells had improved persistence and if they were prone to exhaustion due to stimulation by 2 antigens. To address the question of persistence, we further characterized the BiCisCAR T cells in vivo by measuring the proportion of CAR T cells in mouse spleens 21 days after CAR T cell infusion in our model of NALM6 leukemia cells expressing either GPC2 or CD276. We found that the frequency of BiCisCAR T cells persisting in the spleen was significantly higher than that seen in mice treated with single antigen–targeting CARs (Figure 9, E and F). BiCisCAR T cells also persisted in the highest numbers in the spleen (Figure 9G). To monitor the CAR T cell exhaustion status, we analyzed the expression of the protein markers programmed cell death 1 (PD-1), lymphocyte activation gene 3 (LAG-3), and T cell immunoglobulin and mucin domain–containing protein 3 (TIM-3) on these 3 CAR T cells. Both CD4^+^ and CD8^+^ BiCisCAR T cells expressed the lowest levels of PD-1, LAG-3, and TIM-3 compared with the other 2 CAR T cells targeting single antigens (Figure 9, H–M). Together, these data demonstrated that the BiCisCAR had longer persistence and was less prone to exhaustion in the tumor model with heterogeneous expression of GPC2 and CD276.

To further validate the efficacy of BiCisCAR T cells in a NB heterogeneous model, we used a mixture of 50% IMR5, 25% GPC2-KO IMR5, and 25% CD276-KO IMR5 cells in a metastatic mouse model (Supplemental Figure 12, A and B). Consistent with the NALM6 experiments, BiCisCAR T cells also significantly suppressed the tumor progress compared with single antigen–targeting CAR T cells (Supplemental Figure 12, C and D).

In summary, these experiments demonstrated that the BiCisCAR not only overcame the immune evasion due to heterogenous expression patterns of antigens, but also yielded more potent and persistent T cells with limited exhaustion.

## Discussion

Although several CAR T cell therapies for NB have shown promise in preclinical studies (11, 12, 14, 16, 21, 31, 44), only CAR T cells targeting GD2, L1-CAM, or CD276 have reached clinical trials. CD276 clinical trials are still ongoing (Supplemental Table 1), however clinical trials of GD2 and L1-CAM CAR T cells showed disappointing results, with only a few objective responses seen in the early phase, with the best results showing that 3 of 19 patients achieved a complete response (10–12, 20, 21). However, these preclinical and clinical trials have identified several major challenges that limit the success of using CAR T cells for solid tumor therapy. These include (a) difficulty in identifying tumor-specific antigens; (b) heterogeneous expression of TAAs; (c) CAR T cell exhaustion and limited persistence; (d) difficulty penetrating physical tumor barriers and trafficking to tumor sites; and (e) an immunosuppressive tumor microenvironment for CAR T cells (32, 34).

Optimal NB-associated antigens for CAR T cell therapies are under active investigation, with GD2 and L1-CAM being the most-studied TAAs so far (32). Recent studies by us and others have identified GPC2 and CD276, two cell-surface antigens, as promising targets for CAR T cell immunotherapy against NB (6, 15, 16, 22, 26, 27, 31). GPC2 is specifically and highly expressed on NB tumors, but at a very low level on normal tissues. We have also recently reported potent GPC2-targeting CAR T cells that showed promise in preclinical tests (16). CD276 is overexpressed in many pediatric solid tumors including NBs and tumor blood vessels (22). In addition, the expression of both genes has been reported to predict a more aggressive disease with a poor prognosis (6, 15, 22, 45). These characteristics make GPC2 and CD276 two attractive candidate targets for immunotherapy against NB. In this study, we showed the expression of both targets to be high but heterogenous among NB tumors, predicting that some of the patients with low expression of GPC2 or CD276 would not benefit from CAR T cell therapies targeting either single antigen. Here, we report that 32% of NB tumors expressed low levels of GPC2, although a lower percentage show low mRNA expression of CD276 (18%). However, when mRNA expression of both was combined, 95% of NB samples expressed 1 or both molecules at a high level [log_2_(FPKM_[*GPC2*+*CD276*]_ +1) ≥4]. Hence, dual antigen–targeting BiCisCARs may help a broader cohort of patients compared with single antigen–targeting CARs. Recent IHC studies of NB tumors have also suggested inter- and intratumor heterogeneity of GPC2 and CD276 expression, indicating the potential for selection of antigen-negative clones (6, 35). Thus, targeting two TAA also may offer an advantage to intratumor heterogeneity.

Another challenge of CAR T cell therapies against solid tumors is decreased T cell functionality (32, 34, 46). An optimal CAR should have robust expansion and activity but less exhaustion when exposed to target antigens on tumor cells. Our P-COCC approach was able to identify CAR constructs among a pool of CAR T cells and determine which CARs displayed the highest activity signatures using both transcriptome and protein markers at a single-cell level, an approach allowing an improved identification of cells (39, 47). Combined with expansion dynamics over time and the cytolytic assay, we thus identified 2 optimal CAR constructs against GPC2 and CD276. Therefore, our multimodal approach allows the screening of many CAR constructs against multiple TAAs to find optimal binders. This method eliminates the time-consuming, trial-and-error empirical CAR design and can be applied when multiple CARs need to be evaluated.

To overcome the heterogenous expression of GPC2 and CD276 on NB cells, we developed a bicistronic CAR (BiCisCAR) using 2 of the top CAR constructs identified in this study. This BiCisCAR not only demonstrated effective killing of tumor cells expressing these 2 targets, but also longer persistence and a less exhausted phenotype in vivo. Currently, our GPC2-targeting CAR CT3 is being planned for clinical trial (16), and 6 open CAR T cell clinical trials against CD276^+^ solid tumors including NBs are ongoing (Supplemental Table 1). In addition, the CD276 mAb MGA271, which shares the same VH and VL amino acid sequences with MGB7H3-LH CAR, has been validated to have minimal binding to normal tissues (36) and proven to be safe and well tolerated, with no dose-limiting toxicity in earlier clinical trials (48). Therefore, our BiCisCAR, which incorporated the MGA271 CD276 binder, is likely to have minimal toxicity to healthy organs. The GPC2-targeting CAR, CT3, has density-dependent killing property (16), so it is also likely to have minimal on-target/off-tumor toxicity to normal tissues. However, the single CT3 CAR will have diminished efficacy for NB tumors expressing low levels of GPC2 and limited persistence in vivo. We showed that in the preclinical mouse models, BiCisCARs targeting both GPC2 and CD276 could overcome this uneven expression of GPC2 on NB cells and will be the basis for future trials in humans. Moreover, BiCisCARs exhibited an increased central memory T cell phenotype compared with the MGB7H3LH CAR phenotype, which suggests long-term antitumor properties.

The limitations of our study are that only 14 CAR constructs were analyzed, and we did not determine the maximum number of CAR constructs that could be tested in a single-cell format. The number of CAR T cells detected at 24 hours was reduced, and we attributed this reduction to the use of a CMV promoter, which is prone to be silenced in T cells (49, 50). Work is currently underway to utilize the EF-1α promoter, which may perform better when expressed in T cells. Second, we did not test all the VH-VL combinations, neither did we test all the second- and third-generation CAR T cell construct combinations. In addition, although CAR T cells showed a similar infiltration pattern on day 11 after infusion in a NB PDX model, our current work did not addressed CAR T cell trafficking or the tumor microenvironment such as stromal cells and other suppressive signals, which would reduce the efficacy of CAR T cell therapy against solid tumors. Nevertheless, we were able to identify potent single antigen–targeting CARs against GPC2 and CD276, which showed superior antitumor activity when combined in a BiCisCAR format, including improved cytokine release, longer T cell persistence, and less T cell exhaustion.

In conclusion, we describe a multimodal assay to identify potent candidate CAR constructs within a pool that allow the identification of T cells displaying maximal expansion, activation signatures, and antitumor cytotoxicity. This multimodal approach can be utilized to systematically screen multiple CAR constructs for multiple TAAs to optimize CAR development. Using these methods, we have developed a potent and effective BiCisCAR against NB expressing GPC2 or CD276 that showed prolonged persistence, less exhaustion, and more central memory properties in vivo, and that will be developed as a new adoptive immunotherapeutic for patients with high-risk NB.

## Methods

Additional information can be found in the Supplemental Methods.

### Cell lines and cell culturing

Human NB cell lines, including IMR5, NBEB, SKNAS, NB1691, and IMR32 used in this study were obtained from the NCI Pediatric Oncology Branch (POB). NB1691 and IMR5 cell lines were transduced with lentiviruses expressing the firefly luciferase and GFP (GFP-luciferase) and were obtained from Mitchell Ho at the NCI; and NBEB, IMR32, and IMR5 cells transduced with lentivirus expressing luciferase and mCherry were obtained from Brad St. Croix at the NCI. GPC2- or CD276-KO IMR32 and IMR5 cell lines were generated by CRISPR/Cas9 gene-editing technology and also obtained from Brad St. Croix. The NALM6_GL (GFP-luciferase) cell line was a gift of Haiying Qin from the POB at the NCI.

All of the above-mentioned NB cell lines and NALM6 cells were cultured in RPMI-1640 medium supplemented with 10% FBS, 1% l-glutamine, and 1% penicillin-streptomycin at 37°C in a humidified atmosphere with 5% CO_2_. The Lenti-X-293T lentiviral packaging cell line (Clontech, catalog 632180) was grown in DMEM supplemented with 10% FBS, 1% l-glutamine, and 1% penicillin-streptomycin at 37°C. All cell lines were verified by short tandem repeat (STR) profiling and validated to be mycoplasma-free by MycoAlert (Lonza).

### NALM6_GL cells stably overexpressing GPC2 or CD276

NALM6_GL cells were transfected with the Cell Line Nucleofector Kit T (catalog VCA-1002), Program T-001, Lonza Bioscience), and 2 μg pcDNA3.1-GPC2 or pcDNA3.1-CD276. Transfected cells were then selected by G418 or hygromycin for 7 days individually. The resultant bulk cell populations were separately stained with an anti-GPC2 antibody (clone CT3) or an anti-CD276 antibody (Abcam, clone EPNCIR122), and then sorted into high-expressing cell lines using the FACSAria (BD Biosciences). The bulk cell populations were then single-cell cloned on 96-well plates to create clones with GPC2 or CD276 expression.

### Competition assay to measure expansion dynamics and single-cell profiling

Eight anti-GPC2 and 6 anti-CD276 CAR T cells were first thawed separately, including LH1; LH2; LH3; LH4; LH6; LH7; CT3; G27; m276-HL; m276-LH; MGB7H3-HL; MGB7H3-LH; h8H9-HL; and h8H9-LH and recovered overnight in AIM-V medium (Gibco, Thermo Fisher Scientific) with 40 IU/mL recombinant IL-2 (rIL-2, Clinigen). The next day, CAR T cells were collected and washed twice using RPMI media with 10% FBS to remove rIL-2 individually. IMR5-GL cells (1.6 × 10^6^) were seeded in a 10 cm dish. Then, CAR T cells were pooled in same way as in the counterpart experiment and cocultured with tumor cells using an E/T ratio of 1:1 for individual anti-GPC2 CAR T cells except for G27 CAR T cells, and at an E/T ratio of 1:4 for each anti-CD276 CAR T cell and G27 CAR T cells. Pooled CAR T cells were evenly aliquoted into the designated dishes and cocultured with or without IMR5 cells, a NB *MYCN*-A cell line expressing high levels of GPC2 and CD276, for 1, 2, 3, 5, or 7 days. Suspension cells including CAR T cells and remaining target cells were collected, washed with cold PBS, and pelleted for genomic DNA and RNA extraction every 24 hours and until the seventh day. For the remaining dishes with targets, 1.6 × 10^6^ IMR5_GL cells were added to continuously stimulate CAR T cells every 24 hours until the sixth day.

### ddPCR

Genomic DNA from cells was isolated using the FlexiGene DNA kit (QIAGEN). Digital PCR was performed on a QX200 ddPCR system (Bio-Rad) according to the manufacturer’s instructions. Fourteen CAR-specific primers and probes were multiplexed with a human reference gene (myocardin-like protein 2 [*MKL2*]) assay (Supplemental Table 2). GFP primers were used to calculate all CAR copy numbers, and Luc primers were used for the calculation of copy numbers in IMR5_GL cells. The primers are listed in the Supplemental Table 2.

### Single-cell CITE-Seq and CAR detection using modified TCR-VDJ strategy PCR

Eight anti-GPC2 and 6 anti-CD276 CAR T cells were pooled as described above in a competitive assay. Pooled CAR T cells were evenly divided into 7 fractions for the multimodal single-cell assay. One of the 7 fractions was taken as CTRL_0h, and the remaining 6 fractions were split into 2 groups: 1 group of 3 fractions cultured without target tumor cells for 24 hours (CTRL_24h) and another group of 3 fractions cocultured with target IMR5 cells for 24 hours (STIM_24h). To increase cell viability to greater than 85%, dead cells were removed by magnetic negative selection (Dead Cell Removal Kit, Miltenyi Biotec, catalog 130-090-101). For 2 groups of pooled CAR T cells at 24 hours, TotalSeq-C human “hashtag” antibodies (Supplemental Table 3) were used for each of the CAR T cell triplicates in 2 groups, allowing identification of different samples in the analysis, and then triplicates in each group were combined. Next, 3 CAR T cell pools (CTRL_0h, CTRL_24h, and STIM_24h) were stained with a cocktail of a TotalSeq-C human lyophilized panel (BioLegend) of 31 surface proteins (Supplemental Table 3). After 3 washes, CAR T cells were resuspended in PBS and counted before proceeding immediately to single-cell immune profiling using a 10X Genomics Chromium system. Briefly, T cells were mixed with the reverse transcription (RT) mix and partitioned into single-cell gel-beads in emulsion (GEM) using 10 × 5′ Chromium Single Cell Immune Profiling Next GEM version 1.1 chemistry (10X Genomics). For detection of 14 CAR binders, a fraction of amplified full-length cDNA was used for PCR with CAR-specific reverse primers (Supplemental Table 4), applying a similar approach as that for T cell receptor (TCR-VDJ) sequencing libraries according to the 10X Genomics user’s guides (https://www.10xgenomics.com/resources/user-guides/). 10X Genomics 5′ single-cell gene expression and cell-surface protein tag libraries were prepared as instructed by the 10X Genomics user’s guides. Both libraries and CAR binder libraries were sequenced on an Illumina NextSeq 500 individually.

### CAR binder library sequencing by PacBio-Seq and MiSeq

For CAR binder detection, a fraction of the above amplified full-length cDNA was used for CAR binder enrichment with the custom reverse primers (Supplemental Table 4), and then size selected products (>600 bp) were sequenced by the PacBio Sequel II for full-length binder sequences. The other half of the PCR products without size selection were used for sequencing on an Illumina MiSeq (MiSeq Reagent Kit, version 3; 600 cycles) for CAR binders that were shorter than full length.

### Animal study

Five~ to 8~week-old female NSG mice (NOD.Cg-PrkdcscidIl2rgtm1Wjl/SzJ; NCI CCR Animal Resource Program, NCI Biological Testing Branch) were used for animal experiments.

#### Subcutaneous PDX NB model.

NBEB_Luc-p2a-mCherry– or luciferase-expressing SJNB012407 PDX tumor cells (2 × 10^6^ cells, obtained from St. Jude Children’s Research Hospital, Memphis, Tennessee, USA) were resuspended in Matrigel (Corning) and s.c. injected into 1 flank of each mouse. After tumor establishment, the mice were randomized into 4 groups and separately infused via the tail vein with mock T cells or 2 × 10^6^ CAR T cells once on day 12 for the NBEB model, or with 3 × 10^6^ CAR T cells once on day 14 for the NB PDX model. Tumor volumes were measured by total bioluminescent flux using a Xenogen IVIS Lumina (PerkinElmer) every week. Mice were injected i.p. with 3 mg d-luciferin (PerkinElmer) and imaged 20 minutes later. Living Image software (PerkinElmer) was used to analyze the bioluminescence signal flux for each mouse as photons per second per square centimeter per steradian (photons/s/cm^2^/sr), and tumor volume was measured by caliper and calculated using the formula (length × width^2^)/2. Mice were euthanized when the tumor size reached 4000 mm^3^.

#### 1:1 mixed NALM6-GPC2 and NALM6-CD276 metastatic model.

Luciferase-expressing NALM6-GPC2 (0.5 × 10^6^) and NALM6-CD276 (0.5 × 10^6^) cells were mixed and i.v. injected into NSG mice. Three days later, mock T cells (2.5 × 10^6^ or 5 × 10^6^) of each type of CAR T cell were injected to treat the mice. NALM6 leukemia was detected using the Xenogen IVIS Lumina (PerkinElmer) every 3 or 4 days. Twenty-one days after CAR T cell infusion, mice treated with 5 × 10^6^ CAR T cells were euthanized for spleen collection, and splenic cells were used for flow cytometric analysis of NALM6 cells remaining in the spleen and of T cells phenotype.

#### 2:1:1 mixed IMR5, GPC2-KO IMR5, and CD276-KO IMR5 metastatic model.

Luciferase-expressing IMR5 (1 × 10^6^), IMR5-GPC2–KO (0.5 × 10^6^), and IMR5-CD276–KO (0.5 × 10^6^) cells were mixed and i.v. injected into NSG mice. Fourteen days later, mock T cells or 2 × 10^6^ of the indicated CAR T cells were injected into the mice. Tumor growth was monitored weekly using the Xenogen IVIS Lumina (PerkinElmer).

### Data availability and material transfer agreements

Raw and processed data from the scRNA, surface protein, and CAR binder sequencing experiments are deposited in the NCBI’s Gene Expression Omnibus (GEO) database (GEO GSE181437). Multimodal single-cell analysis code is available at: https://github.com/CCRGeneticsBranch/CITEseq_Screening_CARs Materials created in this study can be obtained under a material transfer agreement. Inquiries should be directed to the corresponding author.

### Statistics

GraphPad Prism software was used for data analysis. Log-rank statistical tests were used to calculate *P* values for survival analyses, an unpaired, 2-tailed Student’s *t* test was used to calculate significant difference between 2 groups, an ordinary 1-way ANOVA was performed for multiple comparisons, and a 2-way ANOVA was used to calculate *P* values for in vitro cytokine production data, in vivo tumor growth curves, and bioluminescence signal analysis. A nonparametric Kruskal-Wallis test was performed to calculate *P* values for CAR T cell counts in the spleen from NALM6 cell–bearing mice among treatment groups. A *P* value of less than 0.05 was considered statistically significant.

### Study approval

All animal studies were approved by the NCI’s Bethesda Animal Care and Use Committee at the NIH (protocol GB-011). Studies using blood from 3 healthy donors were approved by the NIH.

## Author contributions

JK, MT, and JSW designed the experiments. MT, ATC, DM, and JTW performed the experiments. MT collected, analyzed, and interpreted the data. MT, HCC, AA, and X Wen analyzed multimodal single-cell assay data. YKS, DM, JTW, NL, CMD, HQ, ZZ, SB, MB, NT, BSC, and MH provided technical or material support. MCK provided technical support for the CITE-Seq experiment, and MT, HCC, AA, and X Wen analyzed the multimodal single-cell assay data. X Wu provided technical support for the ddPCR experiment. MT wrote the original draft of the manuscript. JK, JSW, MT, MH, BSC, NT, YYK, DM, KEM, ATC, ZZ, and SB reviewed and edited the manuscript.

## Supplementary Material

Supplemental data

## Version 1 07/19/2022

In-Press Preview

## Version 2 08/15/2022

Electronic publication
