# Supplementary material for: An optimized bicistronic chimeric antigen receptor against GPC2 or CD276 overcomes heterogeneous expression in neuroblastoma
Source: J Clin Invest. 2022 Aug 15;132(16):e155621. doi: 10.1172/JCI155621 (PMC9374382; doi:10.1172/JCI155621)
Supplement: Supplemental data [file jci-132-155621-s102.pdf]

Supplementary Materials

Optimizing binders for a bicistronic CAR against GPC2 or CD276 overcoming heterogeneous expression in neuroblastoma

Materials and Methods

Supplemental Figure 1. Correlation of GPC2 or CD276 expression at RNA and protein level.

Supplemental Figure 2. Design and transduction of 14 CAR constructs into T-cells.

Supplemental Figure 3. CAR T-cells identification methods and 14 CAR T-cells profiling by multimodal single cell assay on day 0.

Supplemental Figure 4. Multimodal integration analysis of single cell RNA and protein data from CITE-seq data enhances $CD4^+$ or $CD8^+$ T-cells separation comparing with scRNAseq alone.

Supplemental Figure 5. The metabolic program of CT3 CAR T-cells is skewed towards glycolysis rather than OXPHOS after activation.

Supplemental Figure 6. The numbers of identified CAR T-cells in integrated 24h samples and differential expression assay for CD276 CAR T-cells.

Supplemental Figure 7. BiCisCAR outperforms single CARs *in vitro* killing either GPC2 or CD276 over-expressing NALM6 cells, or GPC2KO or CD276KO NB cells.

Supplemental Figure 8. Dual activation of binders on BiCisCAR shows an additive effect on cytotoxicity cytokines production.

Supplemental Figure 9. MGB7H3LH and BiCisCAR shows comparable efficacy to eliminate GPC2 and CD276 high expressing NBEB in vivo.

Supplemental Figure 10. T-cells infiltration pattern and memory T-cell phenotypes in tumors from NB PDX SJNB012407 subcutaneous model.

Supplemental Figure 11. BiCisCAR can effectively eliminate NALM6 leukemia cells expressing either GPC2 or CD276.

Supplemental Figure 12. BiCisCAR outperforms single CARs in vivo suppressing IMR5 cells heterogeneously expressing GPC2 and CD276.

Note: references 51 ~ 58 are only cited in SM.

Other Supplementary Material for this manuscript includes the following:

Supplemental Table 1. Summary of on-going CAR T cell clinical trials for neuroblastoma.

Supplemental Table 2. CAR specific primers and probes used in ddPCR.

Supplemental Table 3. Cell hashing antibodies and antibody-oligo conjugates directed against T-cell antigens were used in multimodal assay.

Supplemental Table 4. CAR binders specific primers used for enrichment library.

Supplemental Table 5. Top 25 genes list exclusively defining CAR T-cells cluster 11 filtered by fold change > 2, comparing cluster 11 vs other CD8 clusters (0, 2, 4, 5, 6, 9 ,13) of CAR T-cells from the scRNA-seq data.

Supplemental Table 6. Top 20 differentially expressed genes (DEGs) in CT3 CAR T-cells compared with 7 other anti-GPC2 CARs from above DE gene set.

Supplemental Table 7. 14 upregulated expression genes with fold change > 2 in IMR5 stimulated MGB7H3-LH CAR T-cells compared with 5 other anti-CD276 CARs.

MATERIALS AND METHODS

Synthesis of eight GPC2 targeting CAR constructs and six CD276 targeting CAR constructs

Six GPC2 V_{HS} (single-domain antibody variable fragments) obtained through 4-rounds of bio-panning combinational engineered human VH single domain phage display library (15) were introduced into a lentiviral expression vector pLenti6.3/v5(Invitrogen) containing a CD8α hinge and transmembrane domain, a 4-1BB costimulatory motif and a CD3ζ intracellular signaling domain. The resulting GPC2 CARs constructs were separately named as LH1, LH2, LH3, LH4, LH6 and LH7 CAR. CT3, the GPC2 single-chain variable fragment (scFv) isolated from mouse mAbs against GPC2 which was described previously (16) was also cloned into pLenti6.3 or pELPS vector separately to generate two CT3 CAR constructs individually controlled by CMV or EF-1α promoter. Another unreported GPC2 scFv G27 derived from a fully human antibody library, obtained from Dr. Dimiter Dimitrov of Department of Medicine, Division of Infectious Diseases, University of Pittsburgh were cloned to generate G27 CAR construct.

CD276 scFv m276 obtained from Dr. Brad St. Croix (22) were introduced into pLenti6.3 vector to generate m276-HL and m276-LH constructs; CD276 scFv MGA271 provided by MacroGenics (Rockville, MD) were used to construct MGB7H3-HL and MGB7H3-LH CAR (36); CD276 scFv 8H9 provided by Dr. Nai-kong V. Cheung of Memorial Sloan Kettering Cancer Center were used to generate h8H9-HL and h8H9-LH CAR constructs (37).

Synthesis of GPC2/CD276 BiCisCAR

GPC2/CD276 dual targeting CAR was assembled using GPC2- and CD276-binding scFv regions derived from the above validated single antigen targeted CARs, CT3 and MGB7H3-LH. The optimal spacer domain and signaling domain including CD8 HTM, 4-1BB and CD3ζ were selected

for each CAR. We used a cleavable P2A sequence to separate with two CARs constructs so that both can co-expressed upon transduction. Any homologous sequences were codon-wobbled to avoid recombination. Finally, designed GPC2/CD276 BiCisCAR was synthesized followed by cloning into pELPS lentiviral transfer vector with EF-1α promoter.

PBMCs isolation

Peripheral blood mononuclear cells (PBMCs) obtained from blood of healthy donors were isolated using Histopaque®-1.077gm/mL (Sigma, Cat# 10771) according to the manufacturer's instructions.

CAR Lentiviral production and T-cell transduction

The forementioned fourteen CARs or BiCisCAR-encoding lentiviral supernatant was produced by transient transfection of the Lenti-X-293T lentiviral packaging cell line with the corresponding CAR plasmids, using previously described method (51).

Thawed PBMCs were activated with CD3 and CD28 microbeads at a ratio of 1:1 (Dynabeads Human T-Expander CD3/CD28, Thermo Fisher Scientific, Cat# 11141D) in AIM-V media (Invitrogen) containing 40IU/mL recombinant IL-2 (rIL-2, Clinigen Inc.) and 5% heat-inactivated FBS for 48 hours. For each CAR T-cells making, 12 million activated PBMCs were used to be transduced with CAR expressing lentiviral at a multiplicity of infection (MOI) of 14 and resuspended in total 15 mL fresh AIM-V media with 10mg/mL protamine sulfate and 200IU/mL rIL-2 in 6-well plates. T-cells were then centrifuged at 1,000×g for 2 hours at 32°C and incubated overnight at 37°C. A second transduction was performed on the following day by repeating the same transduction procedure as above described. The CD3:CD28 beads were removed on the third day following transduction and transduced T-cells were cultured at 3E5 cells per milliliter in

AIMV medium containing 200IU/mL IL-2, with fresh IL-2-containing media added every 2–3 days until harvest on day 8 or 9. Mock T-cells, also called un-transduced T-cells (UTD) were treated the same as transduced T-cells except during the transduction procedure.

RNA sequencing for GPC2 or CD276 expression on NB tumors or cell lines

Buk RNA-seq libraries preparation, sequencing and analysis pipelines were previously described (52).

Quantitation of GPC2 or CD276 molecules on NB cells by PE quantitation beads

Staining for GPC2 or CD276 expression on patient-derived NB cell lines was performed with mouse anti-human GPC2 antibody (clone CT3, from Dr. Mitchell Ho, NCI) or Rabbit anti human/mouse CD276 antibody (Abcam, clone EPNCIR122), followed by incubation with PE-conjugated Goat anti-mouse immunoglobulin G (IgG) antibody (Biolegend, Cat# 405307) or R-Phycoerythrin AffiniPure F(ab')$_2$ Fragment Goat Anti-Rabbit IgG (H+L) (Jackson ImmunoResearch Laboratories, Cat# 111-116-144). GPC2 or CD276 surface molecules were calculated by the Quantibrite PE Quantitation Kit (BD Biosciences, Cat# 340495) according to the manufacturer's protocol.

CAR T-cell identification analysis

Umi-tools (53) was used to create a whitelist from the scRNA-seq combined read1 fastq file with parameters --set-cell-number=5000 and --set-cell-number=15000 for cell hashed triplicates in addition to --error-correct-threshold=1. Extracting and correcting UMIs and barcodes was done through umi-tools extract function, for PacBio the 26 bp before the Template Switch Oligo (TSO) sequence, for MiSeq and NextSeq first 26 bp in read1. For analysis of CAR binder sequencing data from PacBio, MiSeq or NextSeq, a blast database of the CAR sequences was created in

BLAST+ and blastn was used to align the sequence reads. A custom script was used to deal with multimapping by selecting best hit for each read using the criterion of disregarding reads mapped to more than one CAR with same accuracy level. If different UMIs from same barcode (cell) are mapped to different CARs, they will be removed as well.

After separately obtaining a final matrix containing reads counts per cell from Pacbio-Seq, MiSeq, or NextSeq, another custom script was used to filter cells with less than three reads and combine above three matrices to get a final matrix containing cells with unique CAR annotation.

Single cell data processing

CellRanger (10x Genomics) version 3.1.0 was used to map to the human genome (version GRCh38) and to count antibody tag features. Data were further processed using Seurat (v.3.1.0 or 4.0.0) (54) running in R v4.0.5. After loading in raw matrix of single cells protein and RNA expression, we further demultiplexed the triplicates using the hashtag antibodies staining using Seurat's HTOdemux function. Cells with less than 300 or greater than 5,000 detected genes, greater than 25% mitochondrial reads were filtered.

Single cell RNA-seq data integration

scRNA-seq data from sample CRTL_24h or STIM_24h were first analyzed through standard pipelines in Seurat which include normalization and feature selection. To perform integration, repeatedly variable features across two datasets were selected, anchors were identified by the 'FindIntegrationAnchors' function with 1:60 dimensions and 2,000 anchor features, and then two datasets were integrated together through these anchors with 'IntegrateData' function. For downstream analysis of integrated assay, standard workflow including data feature scaling (ScaleData), principal-component analysis (PCA; RunPCA) was performed and called as

‘integrated_pca’. A SCT assay was also generated using sctransform based normalization which enables recovering sharper biological distinction compared to log-normalization (55).

Denoised and Scaled by Background (DSB) normalization of single cell protein data

Single-cell protein data (representing the quantification of antibody-derived tags (ADTs) in CITE-seq data) was normalized by the DSB method (56), which removes technical noise associated with unbound antibody. The following parameters denoise_counts =TRUE and use.isotype.control =TRUE were used in the dsb normalization function, which models and regresses out a covariate corresponding to the technical component of the cell’s protein library by combining the per cell background and isotype control counts.

Single-cell cell surface protein level expression: DSB normalization matrices of two cultured samples were separately added into the above ADT assay of Seurat objects as normalized data. All measured proteins excluding isotype control were set up as variable features. Subsequently, PCA was performed in ‘prcomp’ function in R due to DSB normalized data cannot be scaled again. Then this custom dimensional reduction was stored as 'pca_adt' into combined Seurat object with integrated RNA assay and ADT assay.

Multimodal weighted nearest-neighbors (WNN) analysis

After pre-processing and dimensional reduction on both integrated RNA and ADT assays independently, weighted nearest neighbors (WNN) analysis was performed in a function ‘FindMultiModalNeighbors’, provided by Seurat v4.0.0-beta (47). Then a UMAP reduction was obtained based on the above WNN graph, and cells were clustered using ‘FindClusters’ function in Seurat with resolution = 0.8. A total of 16 clusters were generated and defined by common markers. And differential expressed genes across clusters were identified using the ‘FindMarkers’

function. Cluster 14 is a subgroup with high expression of mitochondrial gene, which was excluded from subsequent analysis.

Differential Expression (DE) Analysis of genes expression

The differential expression genes across condition or CAR T-cells were performed using the 'FindMarkers' function in Seurat with parameter logfc.threshold =0, which is based on the non-parametric Wilcoxon rank sum test. DE assay was performed between cluster 11 and other $CD8^+$ clusters (0, 2, 4, 5, 6, 9, 13) to identify the genes list specifically expressed on cluster 11. When comparing stimulated CT3 CAR T-cells with seven other GPC2 targeting CARs, a SCTransform (SCT) assay was used in differential expression analysis to detect the genes list highly expressed on CT3 CAR. Comparing stimulated MGB7H3-LH CAR and five other CD276 CARs used the same assay and function as above.

Ingenuity Pathway Analysis (IPA)

IPA (QIAGEN) was used to exploit the canonical pathways correlated with each CD8 cluster or CT3, MGB7H3-LH CAR T-cells (57). Core expression analysis was performed using differential expression genes (DEGs) identified in each cluster or CAR T-cell types and their corresponding fold change values. Identified top canonical pathways were used to represent the major molecular and cellular functional profiles of the specific cluster or CAR T-cell type. The z score was used to determine activation or inhibition level of a specific pathway ($z < 0$, inhibited; $z > 0$, activated; $z >= 2$ or $z <= -2$ can be considered significant).

Enrichment of effector T-cell signatures in DE genes

Enriched gene sets were identified using the pre-ranked gene-set enrichment analysis (GSEA) algorithm, ranking by $\log_2$ (fold change). Analysis was performed using the desktop GSEA (v4.1).

ImmuneSigDB gene sets (58) were used to identify enriched gene set significantly expressing difference (FDR<0.05).

Metabolic analyses of CAR T-cells

CAR T-cells were incubated in the absence of target cells or in the presence of IMR5 cells at an E:T ratio of 1:1 for 24 hours at 37˚C. Oxygen consumption rates and extracellular acidification rates were then measured in a Seahorse XFe96 analyzer (Seahorse Bioscience). CAR T-cells (2.5E5) were seeded into 96-well seahorse XF-96 assay plates in Seahorse BASE medium (6 replicates for each condition) and incubated at 37˚C in a non-CO_2 incubator for 45min. Cells were monitored in basal conditions and in response to oligomycin (1 µM; Sigma-Aldrich), FCCP (1 µM; Sigma-Aldrich), rotenone (100 nM, Sigma-Aldrich) and antimycin A (1 µM; Sigma-Aldrich), according to the manufacturer's protocol. OCR and ECAR were automatically calculated and recorded using the Seahorse XF-96 software.

Cytotoxicity Assay of CAR T-cells

(1) **For luciferase assay and cytokines release measurement**, CAR T-cells were co-incubated with 1E4 of target neuroblastoma cells expressing luciferase at different E: T ratios for 20 h. Then supernatant was collected, and cytokines production was measured by a Human Proinflammatory Panel V-plex Human Tissue Culture multiplex assay (Meso Scale Discovery). The remaining tumor cells were lysed and the luciferase activity in the lysates was accessed using the Steady Glo luciferase assay system on Victor (PerkinElmer). Results were analyzed as specific lysis percent based on luciferase activity in wells with tumor cells alone: % specific lysis =100 − [relative light units (RLU) from wells with effector and target cells] / (RLU from wells with target cells) × 100.

(2) **For IncuCyte assay**, 1E5 GFP expressing NALM6, NALM6-GPC2 or NALM6-CD276 in 100µL RPMI media were loaded into a 96-well plate (Corning BioCoat Poly-L-Lysine 96-Well Clear TC-Treated Flat Bottom Assay Plate) individually. Then 100µL of CAR T-cells was added into the designated well on the next day at an E: T ratio of 1:1. The plate was scanned for the GFP fluorescent expression to monitor live target cells by an IncuCyte ZOOM system every 2 hour for a duration of 48 hour. The percentage of remaining tumor cells at each time point was calculated by dividing the total green fluorescence intensity at every time point by the same measurement at the first time point.

(3) **For Cytolytic Assays by xCELLigence® real time cell analysis (RTCA)**, 5E3 target human Neuroblastoma cell lines (NBEB_luc, IMR5_GL and NB1691_Luc) or 5E4 GPC2KO or CD276KO IMR32 or IMR5 cells were separately seeded into an E-Plate 16 (ACEA biosciences). After settling down for 4h, effector CAR T-cells were added into the corresponding wells at an E: T ratio of 1:1. Then the E-plate 96 was placed back to the xCELLigence RTCA SP, and impedance measurements were recorded every 15 min for about 20 additional hours at 37˚C and 5% CO_2. CAR T-cell-mediated death of tumor cells was monitored in real time and was indicated by a decrease in cell index. Data were analyzed with RTCA Software 2.0 (Acea Biosciences). Results were normalized before CAR T-cells addition (about 4h after tumor cells addition).

Activation of BiCisCAR T-cells for intracellular cytokine staining

CAR T-cells were co-incubated with no target, IMR5, IMR5-GPC2KO or IMR5-CD276KO cells at an E: T ratio of 1:1 for 16 hours in 37˚C in an 5% CO_2 incubator. Protein transport inhibitor monensin solution (1000×, Biolegend, Cat# 420701) was then added into each well for another 4 hours, blocking export. Activated CAR T-cells cultured in the indicated conditions were collected for CAR surface staining firstly by GPC2-Fc (Acro Biosystem, Cat# GP2-H5255) or Biotinylated

CD276 protein (Acro Biosystem, Cat# B73-H82F5), subsequently staining with anti-human IgG Fc or streptavidin respectively. IC fixation buffer and Permeabilization Buffer was used to fix and permeabilize the cells after surface staining. Finally, intracellular cytokines were detected using a PE-conjugated anti-human IFN-γ antibody (Clone 4S.B3), Brilliant Violet 650™ anti-human IL-2 antibody (Clone MQ1-17H12) and PerCP/Cyanine5.5 anti-human TNF-α antibody (Clone MAb11) from BioLegend.

Antibodies and flow cytometry analysis

CAR expression on transduced T-cells was measured by flow cytometry. The transduction efficiency of eight anti-GPC2 CAR T-cells or six anti-CD276 CAR T-cells derived from constructs using plenti6.3 vector can be indicated by GFP expression. CT3, MGB7H3-LH, GPC2/CD276 BiCisCAR T-cells derived from constructs using pELPS vector were stained with Biotin-Protein L (Thermo Fisher Scientific, Cat# PI29997), followed by incubation with R-Phycoerythrin-conjugated streptavidin (Jackson ImmunoResearch Laboratories, Cat# 016-110-084). Surface expression of GPC2 targeting (CT3) CAR transduced T-cells was measured by staining with recombinant human GPC2-Fc Chimera Protein (Acro Biosystem, Cat# GP2-H5255), following by AF647-(Fab)$_2$ specific for human IgG-Fc (Jackson ImmunoResearch Laboratories, Cat# 109-607-008). Surface expression of CD276 targeting (MGB7H3-LH) CAR transduced T-cells was detected by staining with Biotinylated Human B7-H3 (4Ig) / B7-H3b Protein (Acro Biosystem, Cat# B73-H82F5), followed by incubation with R-Phycoerythrin-conjugated streptavidin. Expression of BiCisCAR T-cells was assessed using a combination of both detection reagents as indicated for individual figures.

For the T-cell exhaustion and phenotyping panel analysis, T-cells were detected using the following anti-human antibodies: CD45-FITC (BioLegend, Clone HI30), CD3-PE (BioLegend,

Clone HIT3a), Tim-3-PE-Cy7 (BioLegend, Clone F38-3E2), CD4-PE-Dazzle594 (BioLegend, Clone A161A1), CD8-APC (BioLegend, Clone RPA-T8), ICOS-AF700 (BioLegend, Clone C398.4A), CD137-APC-Cy7 (BioLegend, Clone 4B4-1), LAG3-BV510 (BioLegend, Clone 11C3C65), PD-1-BV711 (BioLegend, Clone EH12.2H7), CD62L-Percp/Cy5.5 (BioLegend, Clone DREG-56), CD45RA- Brilliant Violet 605 (BioLegend, Clone HI100). The absolute CAR T-cell counts in the spleen from tumor bearing NSG mice were measured by using CountBright™ absolute counting beads for flow cytometry (Invitrogen, Cat# C36995) according to the manufacturer's protocol.

All staining was performed in 0.1 mL FACS buffer (PBS+ 0.5% BSA+2mM EDTA). Flow cytometry was performed using a FACS Fortessa (BD Biosciences) and analyzed with FlowJo software (Tree Star).

H&E and Immunohistochemistry (IHC) staining T-cell infiltration in tumors

NB PDX Tumors were dissected from mice at day 11 post CAR T-cells infusion and then fixed in 10% NBF for 24 hours. Formalin fixed, paraffin embedded (FFPE) tissue was sectioned at 5 µm. Sections of neoplastic masses contained minimal adjacent normal tissues, including fibroadipose connective tissue and skeletal muscle. IHC staining was performed using anti-human CD4 antibody (Abcam, Cat# Ab133616) and anti-human CD8 antibody (Abcam, Cat# Ab101500) to identify T cells in tumors. Positive IHC controls included human tonsil. Negative controls included replacing the primary antibody with nonspecific antibody from the same species and of the same isotype. Positive and negative controls stained appropriately. Whole Slide Imaging (WSI) was performed with an Aperio ScanScope XT (Leica) at 200× in a single z-plane.

SUPPLEMENTAL FIGURES

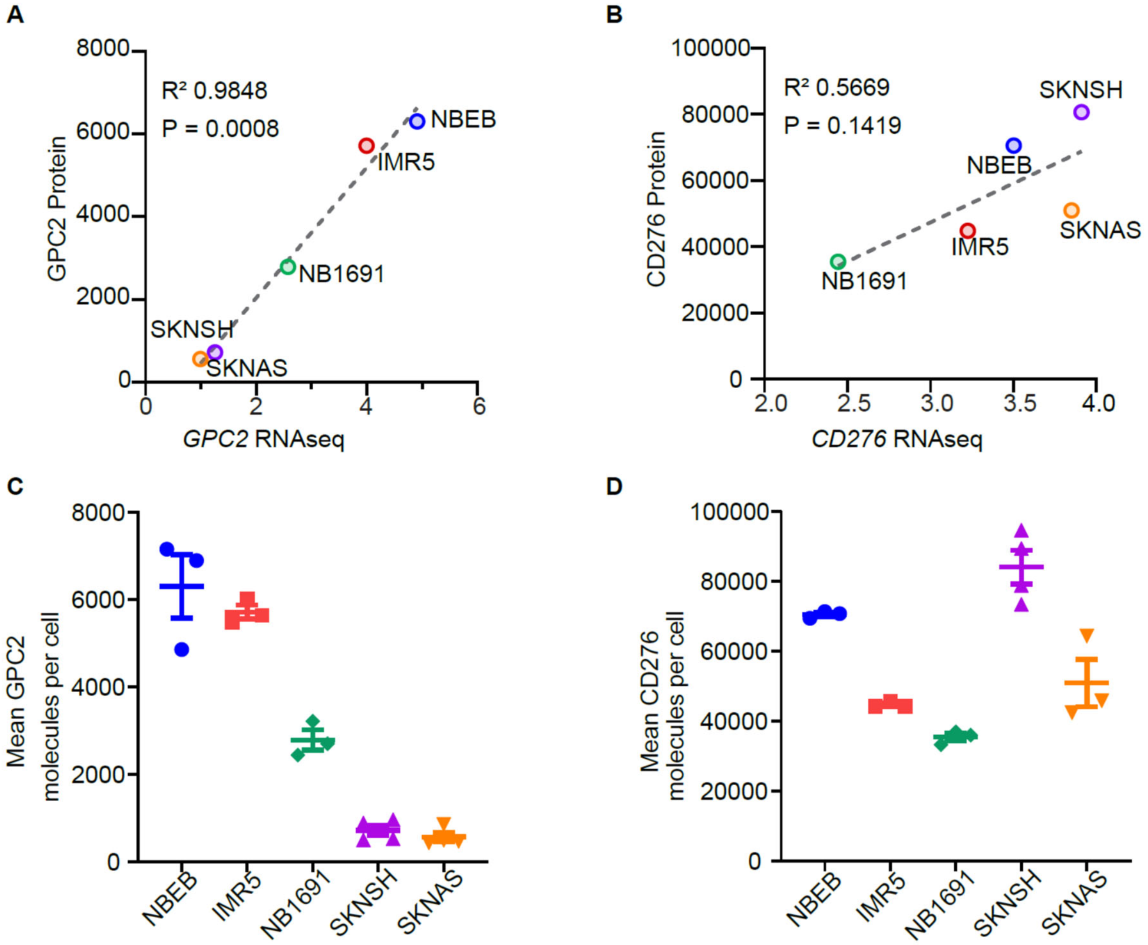

Supplemental Figure 1

Correlation of GPC2 or CD276 expression at RNA to the protein level. (**A**) *GPC2* RNA expression on patient-derived NB cell lines shows high correlation with GPC2 protein level. (**B**) CD276 RNA expression on NB cell lines shows correlation with CD276 protein level. (**C** and **D**) Quantification of GPC2 or CD276 molecules expressed on NB cell lines as determined by PE fluorescence quantitation kit. Data are shown as individual values and means ± SD, *n* = 3 or 4 independent experiments.

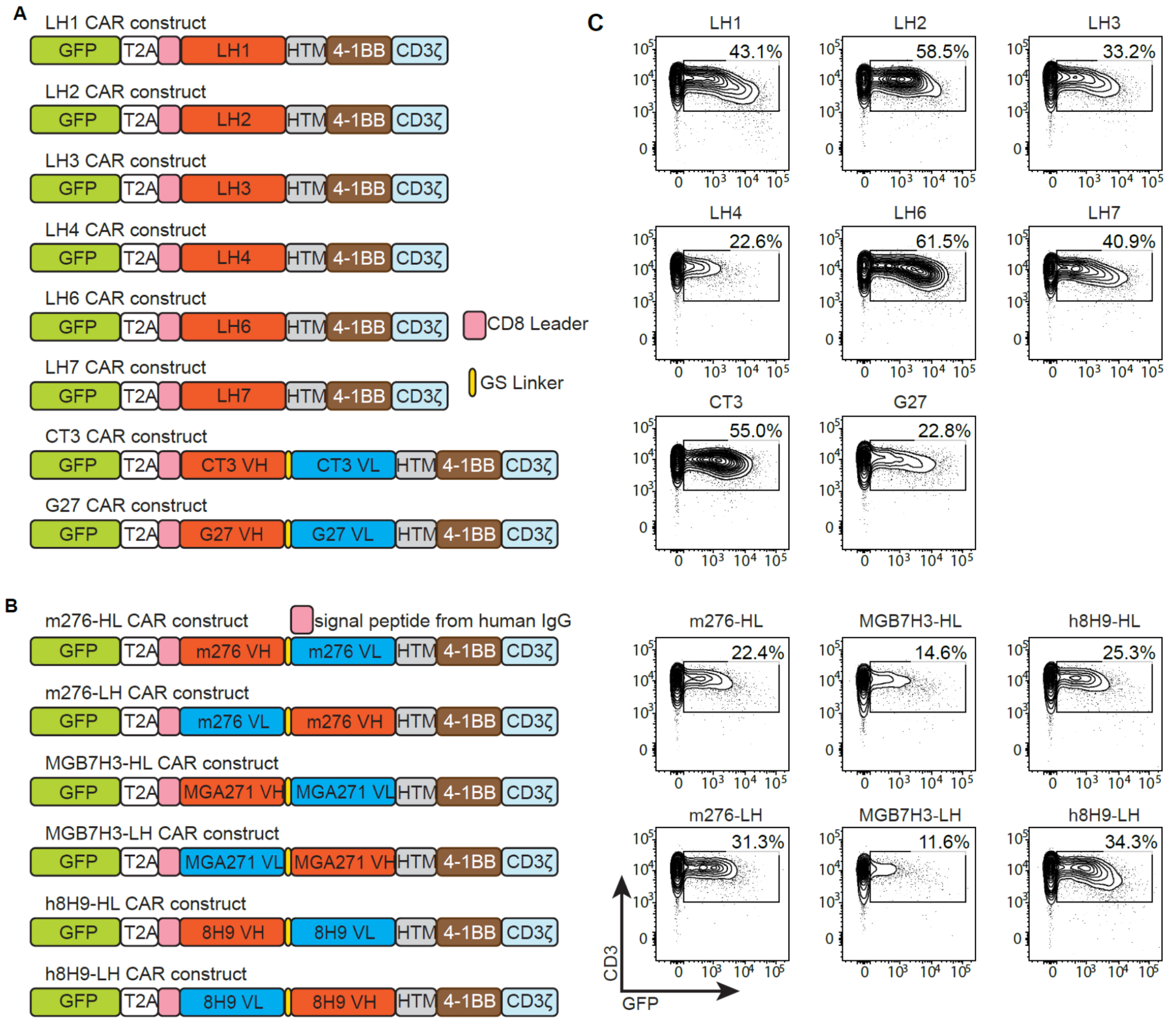

Supplemental Figure 2

Design and transduction of 14 CAR constructs into T-cells. (**A**) Schema of a second-generation lentiviral CAR construct using to individually design 8 GPC2 targeting CARs. Lentiviral constructs express a GFP followed by a T2A ribosomal skipping sequence for protease cleavage. The CAR molecules have a CD8α leader before a CD8α hinge and transmembrane domain, followed by a 4-1BB costimulatory signaling moiety and the cytoplasmic component of CD3ζ signaling molecule. (**B**) Vector maps of 6 CD276 targeting CAR constructs (HTM, hinge and transmembrane domain). (**C**) Representative flow-cytometry plots separately showing 14 CAR constructs surface expression on T-cells before pooling.

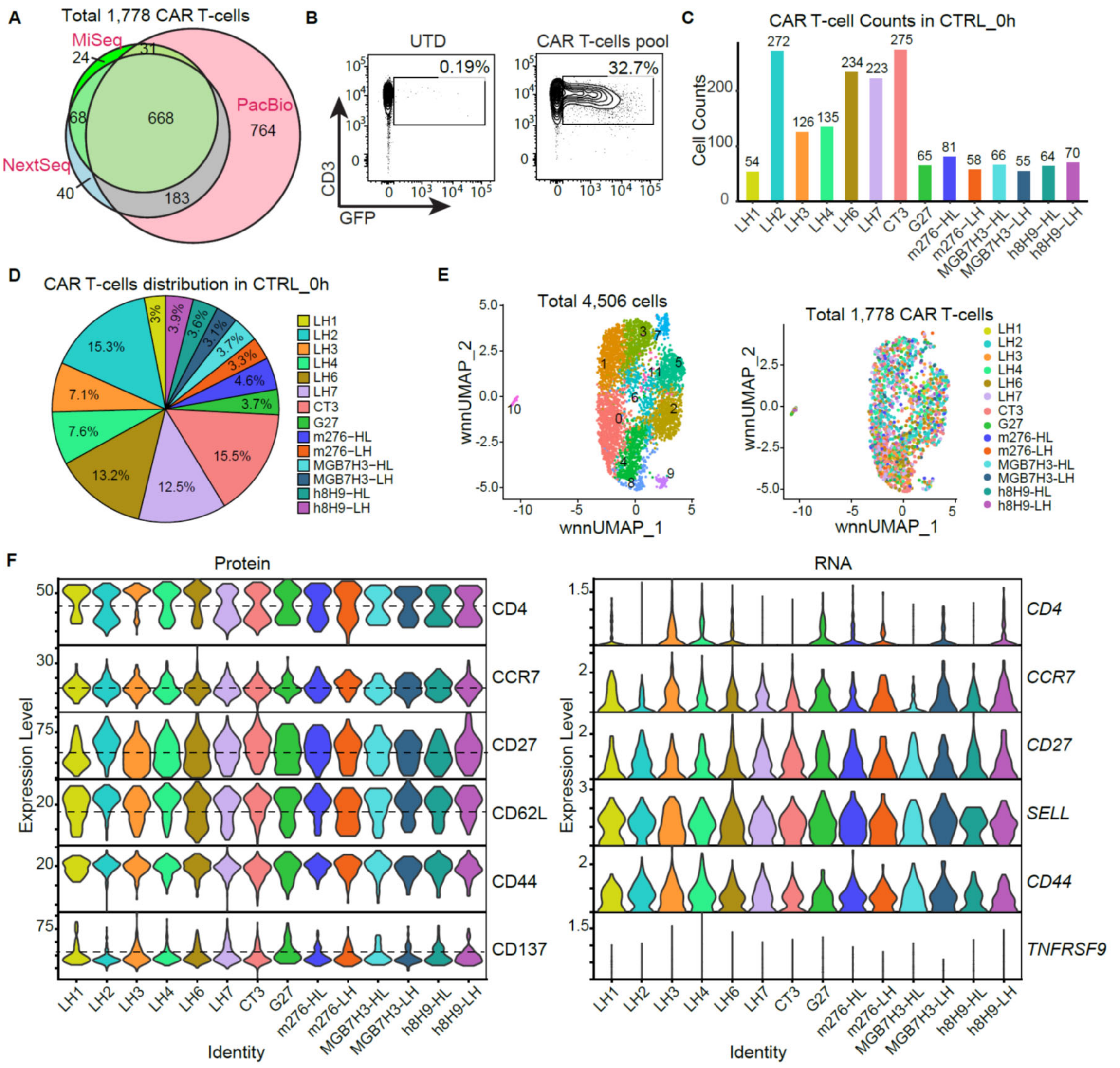

Supplemental Figure 3

CAR T-cells identification methods and 14 CAR T-cells profiling by multimodal single cell assay on day 0. (**A**) Venn diagram showing identified 1,778 CAR T-cells and overlapped counts among PacBio-seq, MiSeq and NextSeq in CAR T-cell pools. (**B**) Flow cytometry plots showing the frequency of CAR expression in T-cells after 14 CAR T-cell pooling indicated by GFP expression. (**C**) Bar plot showing the numbers of each CAR T-cell within identified CAR T-cells of sample CTRL_0h. (**D**) Pie chart showing the proportion of each CAR construct within CAR T-cells pool. (**E**) wnnUMAP visualization of total 4,506 T-cells distributing into 12 clusters (left) and 1,778 CAR T-cells(right) from sample CTRL_0h. (**F**) Protein expression (left) of canonical naïve or activated T-cell markers on identified 14 CAR types from CITE-seq assay. Violin plots (right) showing corresponding RNA expression patterns for *CD4*, *SELL*, *CD44*, *CCR7*, *CD27* and *TNFRSF9* genes in scRNA-seq assay. Cells are grouped by CAR T-cell types identified by three methods in **A**.

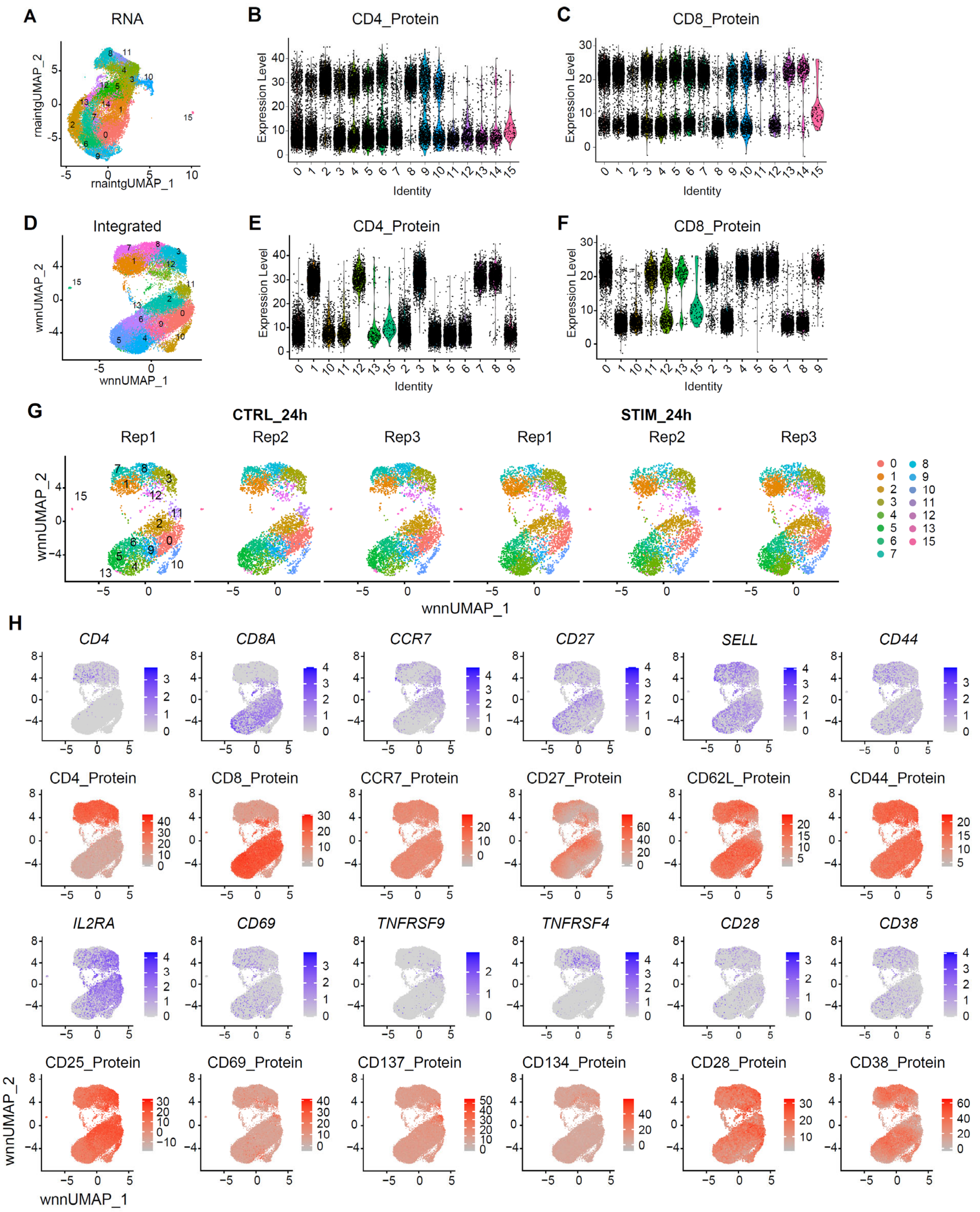

Supplemental Figure 4

Multimodal integration analysis of single cell RNA and protein data from CITE-seq data enhances CD4⁺ or CD8⁺ T-cells separation comparing with scRNAseq alone. (A-C)

Transcriptome-based clustering of single cells reveals that $CD8^{+}$ and $CD4^{+}$ T cells were partially blended when visualization of CD4 (**B**) and CD8 (**C**) protein expression on 15 clusters (A). (**D-F**) UMAP visualization of single cells from integrated CTRL_24h and STIM_24h samples based on WNN assay revealed CD4 or CD8 T-cells were separated clearly in the protein data. Cells are colored according to clusters. Violin plot of the canonical T-cell markers CD4 (**E**) and CD8 (**F**) protein expression on above 15 clusters (**D**). (**G**) UMAP visualization of single cells from integrated sample, split by cell hashing features to identify replicates within two samples. (**H**) mRNA (blue) from transcriptome data and corresponding protein (red) signal from the CITE-seq antibody panel projected on the wnnUMAP plot from panel **D** enhances the characterization potency of T-cells phenotypes.

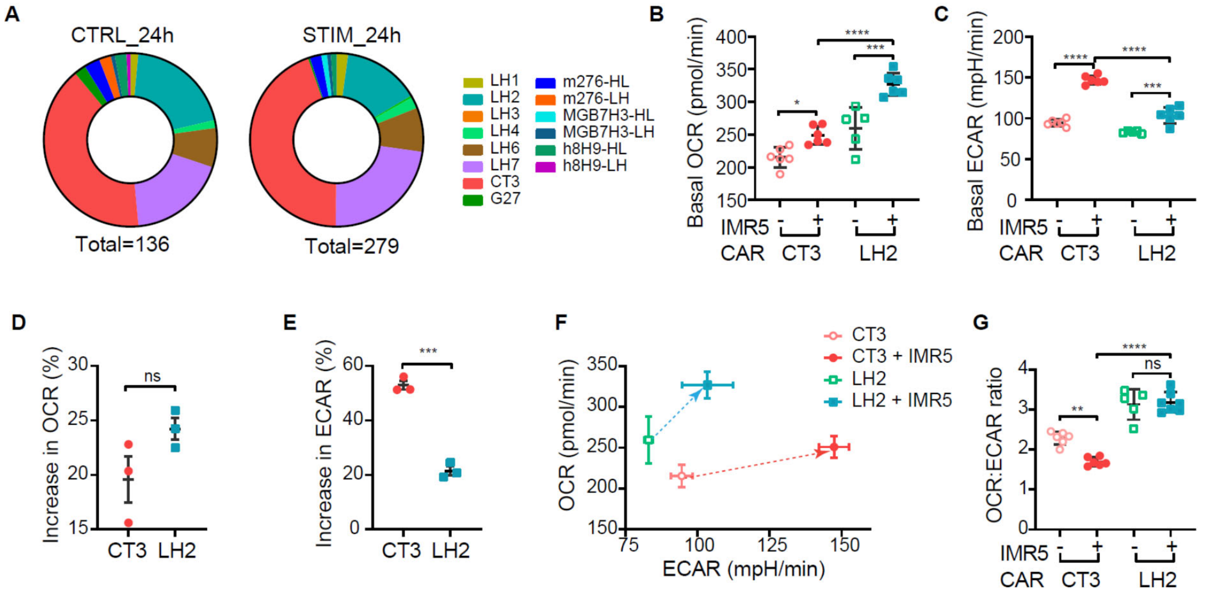

Supplemental Figure 5

The metabolic program of CT3 CAR T-cells is skewed towards glycolysis rather than OXPHOS after activation. (**A**) The distribution of 14 CAR T-cells in cluster 11 from integrated CITE-seq data shows CT3 CAR T-cells to be the dominant CAR, while LH2 with a lower fraction of cluster 11 CAR T-cells. (**B** and **C**) Oxygen consumption rate (OCR, **B**) and basal extracellular acidification rate (ECAR, **C**), a measure of glycolysis, were measured using Seahorse technology for two CAR T-cells with comparable transduction efficiency, CT3 and LH2. Following 24h of coculture with or without IMR5 cells, OCR and ECAR were assayed on a Seahorse XF-96 analyzer following sequential injection of oligomycin, FCCP and Rotenone/Antimycin A and means ± SD are shown ($n = 6$ replicates). CT3 CAR T-cells exhibited a lower OCR and a higher ECAR than LH2 CAR T-cells. Statistical analysis performs with one-way ANOVA, Tukey's multiple comparisons test; *$p < 0.05$, **$p < 0.01$, ***$p < 0.001$, ****$p < 0.0001$. (**D and E**) The percentage of increase in basal OCR (**D**) and ECAR (**E**) following activation of CAR T-cells with IMR5 target cells are presented comparing to the non-stimulated CAR T-cells ($n = 3$ time points). Statistical analyses are performed by unpaired two-tailed t-test. (**F**) OCR/ECAR energy plots (in means ± SD) showed that CT3 CAR T-cells had a significantly higher increase in ECAR than LH2 CAR T-cells followed by tumor cell stimulation. (**G**) CT3 CAR T-cells showed a lower OCR/ECAR ratio ($p < 0.0001$) in the presence of IMR5 target cells. Data are shown in means ± SD from 1 representative experiment. *P* values are determined by one-way ANOVA, Tukey's multiple comparisons test; **$p < 0.01$, ****$p < 0.0001$; not significant, ns.

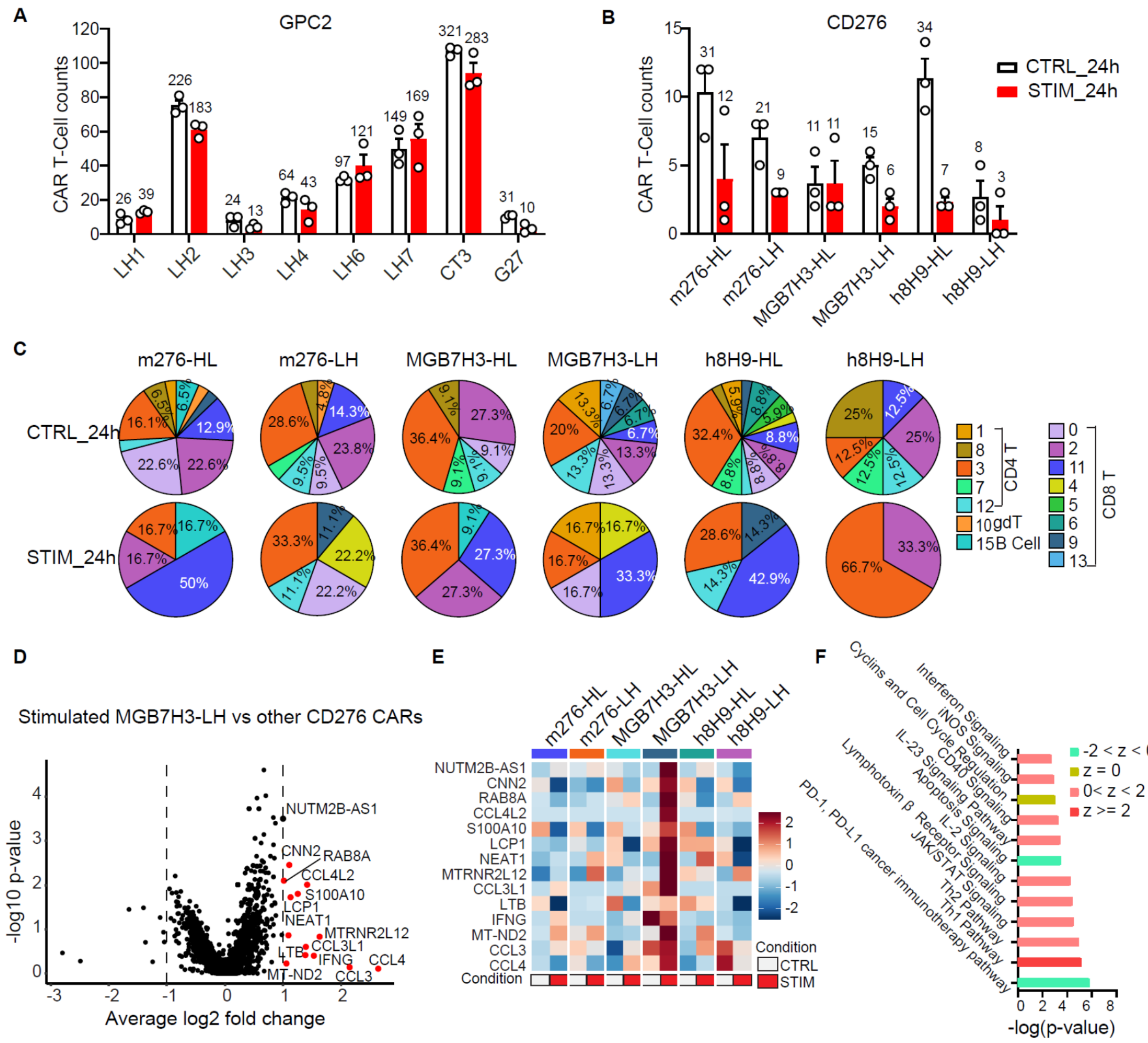

Supplemental Figure 6

The numbers of identified CAR T-cells in integrated 24h samples and differential expression assay for CD276 CAR T-cells. (**A** and **B**) Bar plot showing the numbers of each GPC2 targeting CAR T-cell (**A**) or each anti-CD276 CAR T-cells (**B**) within identified CAR T-cells of CTRL_24h and STIM_24h samples. The total CAR T-cell counts of each construct are labeled on the top of bars from different conditions. The dots mean the 3 replicates of each condition. (**C**) Pie Charts showing the percentage of each cluster within 6 distinct anti-CD276 CAR T-cells. (**D**) Volcano plot of DEGs between MGB7H3LH CAR and 5 other CD276 targeting CARs at 24h post-coculture with IMR5 cells. Top 14 genes ranked by average log2 fold change are colored red. (**E**) Heatmap showing average expression of top14 genes (fold change >2) in 6 CD276 targeting CARs within CAR T-cell pools sample co-cultured with or without targets for 24h. (**F**) IPA identified canonical signaling pathways regulated by DEGs identified in **D**.

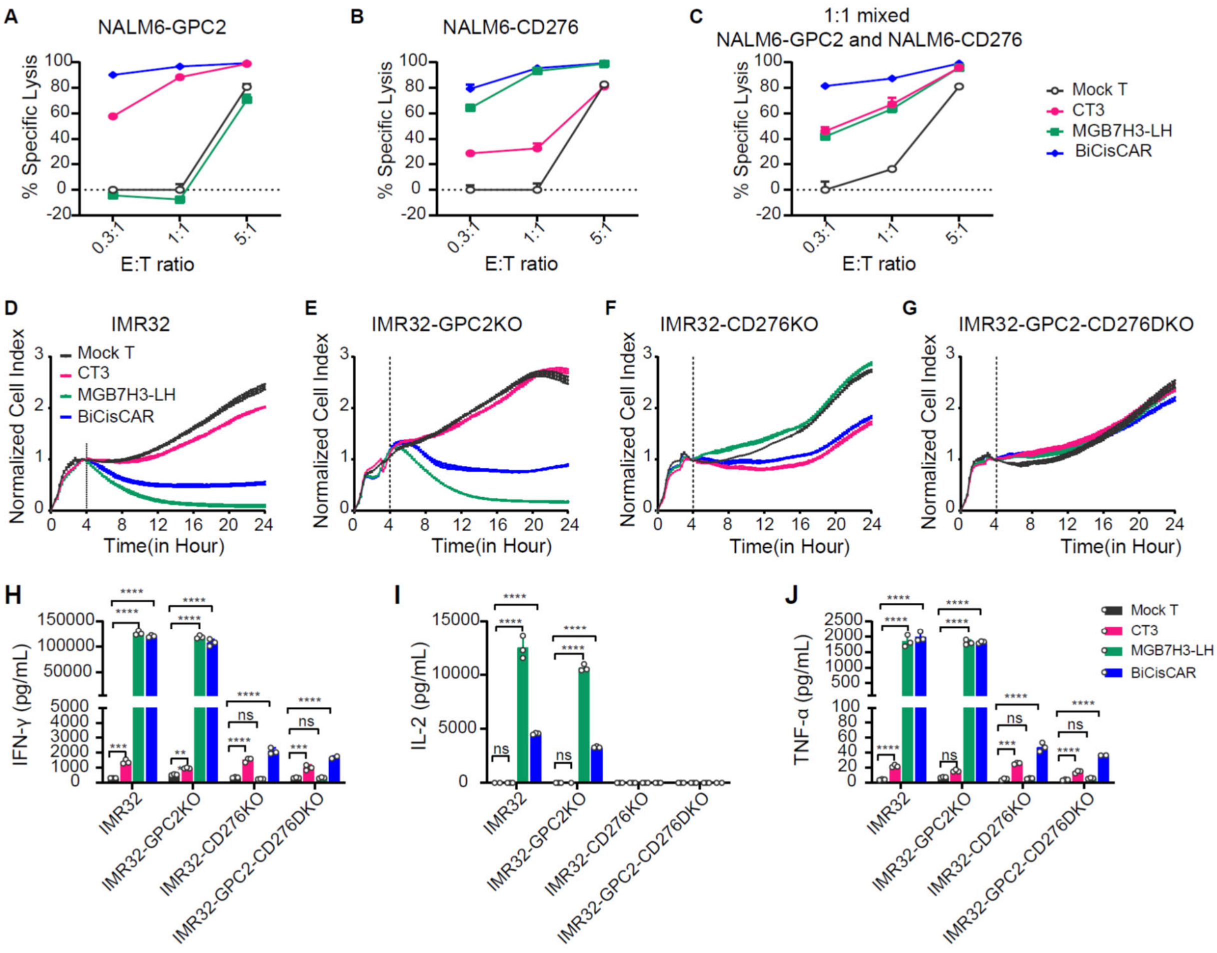

Supplemental Figure 7

BiCisCAR outperforms single CARs *in vitro* killing either GPC2 or CD276 over-expressing NALM6 cells, and GPC2KO or CD276KO NB cells. (**A** to **C**) Luciferase expressing NALM6-GPC2 clone (**A**), NALM6-CD276 clone (**B**) or 1:1 mixed NALM6-GPC2 and NALM6-CD276 cells (**C**) are co-cultured with single antigen targeting or GPC2/CD276 BiCisCAR T-cells for 20h at indicated E:T ratio. And then the specific lysis percentages of tumor cells are evaluated by luciferase assay. (**D** to **G**) Cytotoxicity of single antigen targeting CARs or GPC2/CD276 BiCisCAR after co-cultured with IMR32(**D**), IMR32 cells with CRISPR/CAS9 knockout of either GPC2 (**E**), CD276 (**F**) or both antigens (**G**) at an E:T ratio of 1:1 by ACEA assay. (**H** to **J**). IFN-γ (**H**), IL-2 (**I**) and TNF-α (**J**) production of Mock, CT3 CAR, MGB7H3-LH CAR and GPC2/CD276 BiCisCAR T-cells after 20h of co-culture with the indicated IMR32 cell lines. Data are shown as individual values and the means ± SD, $n = 3$ independent co-culture with CAR T-cells. Statistical analysis represents two-way ANOVA, Tukey's multiple comparisons test in Figure **F** to **H**; ***$p < 0.001$; ****$p < 0.0001$; not significant, ns.

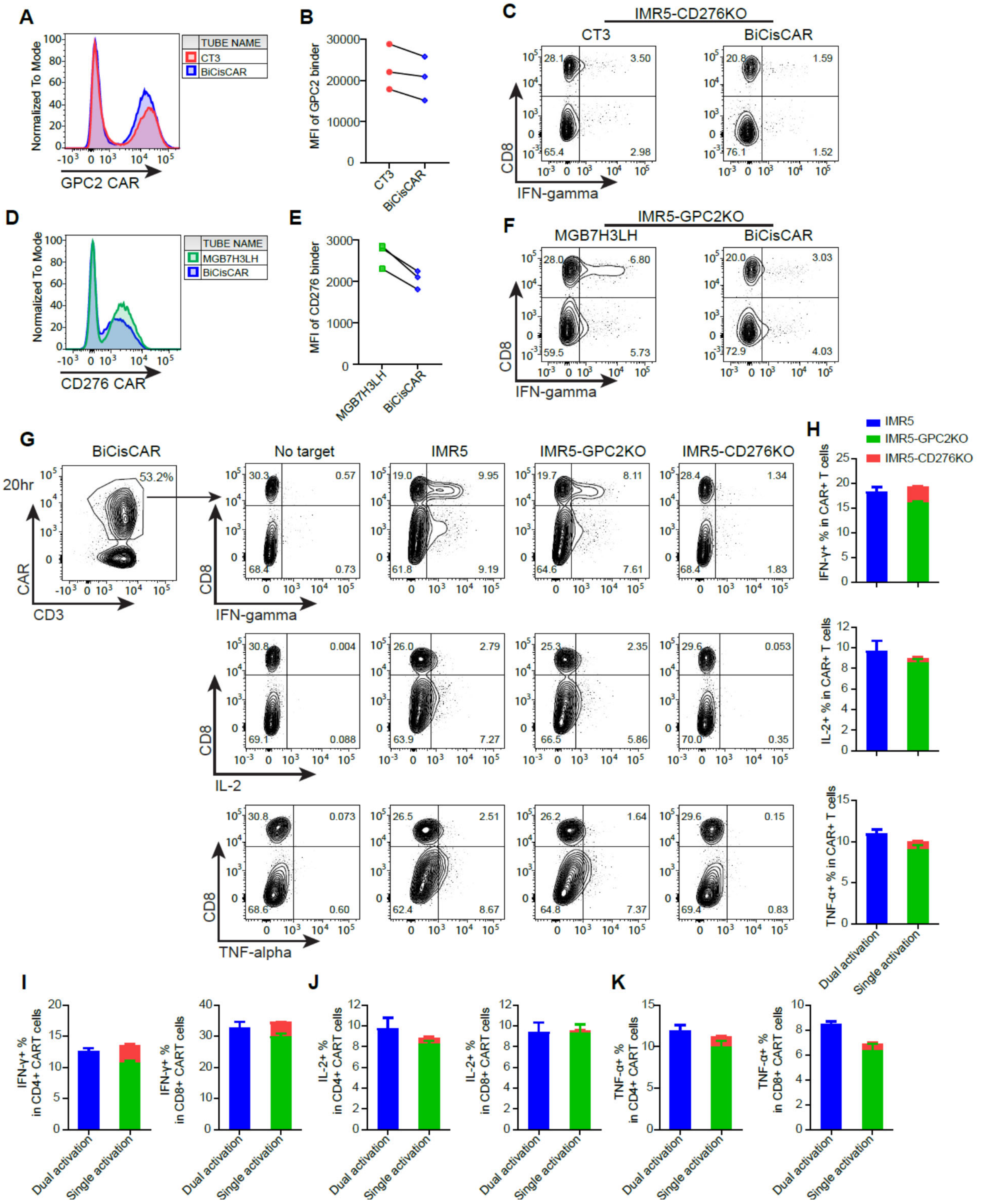

Supplemental Figure 8

Dual activation of binders on BiCisCAR shows an additive effect on cytokine production. (**A** and **B**) GPC2 specific binder expression in CT3 CAR T-cells (red) or BiCisCAR T-cells (blue)

was determined by GPC2-Fc protein staining and subsequently stained with anti-human IgG Fc secondary antibody(**A**). The mean fluorescence intensity (MFI) of CAR expression shows fewer GPC2 binders in BiCisCAR than that of CT3 CAR T-cells (**B**). (**C**) Representative flow-cytometric plots demonstrating the percentage of IFN-γ producing CAR T-cells after GPC2 binder activation by IMR5-CD276KO cells for 6 hours. (**D** and **E**) CD276 specific binder expression in MGB7H3LH CAR T-cells (green) or BiCisCAR T-cells (blue) was determined by biotinylated CD276 protein staining (**D**). The mean fluorescence intensity (MFI) of CAR expression shows less CD276 binders in BiCisCAR than that of MGB7H3LH CAR T-cells (**E**). (**F**) Representative flow-cytometric plots demonstrating the percentage of IFN-γ producing CAR T-cells after CD276 binder activation by IMR5-GPC2KO cells for 6 hours. (**G**) Representative flow-cytometric plots demonstrating IFN-γ, IL-2, or TNF-α production by BiCisCAR T-cells after dual or single activation by IMR5, IMR5-GPC2KO or IMR5-CD276KO for 20 hours. (**H**) The percentage of IFN-γ, IL-2, or TNF-α producing BiCisCAR T-cells after single or dual activation by individual target cells. (**I** to **K**) The percentage of IFN-γ (**I**), IL-2 (**J**), or TNF-α (**K**) production in $CD4^+$ or $CD8^+$ BiCisCAR T-cells after single or dual activation by respective target cells.

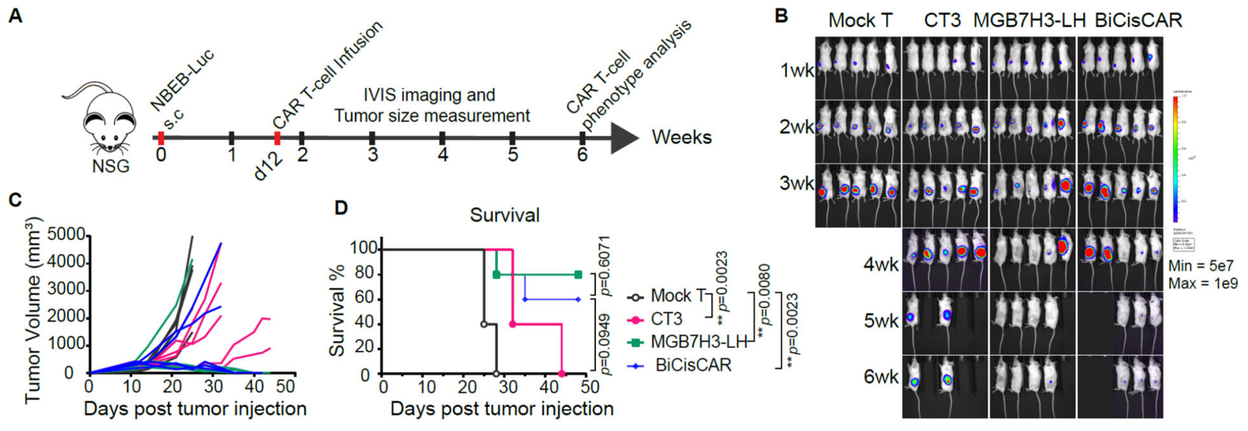

Supplemental Figure 9

MGB7H3LH and BiCisCAR shows comparable efficacy to eliminate GPC2 and CD276 high expressing NBEB *in vivo*. (**A**) Schema of the neuroblastoma subcutaneous xenograft model infused with CAR T-cells on day 12 after tumor inoculation. (**B**) Representative bioluminescence images of NBEB_Luc tumor growth in the subcutaneous model shown in **A**. (**C**) Tumor volumes are measured by caliper. Values for individual mice are shown. (**D**) Kaplan-Meier survival analysis of mice treated with CAR T-cells are shown (5 mice/group). Statistical analysis for survival curves represents log-rank test.

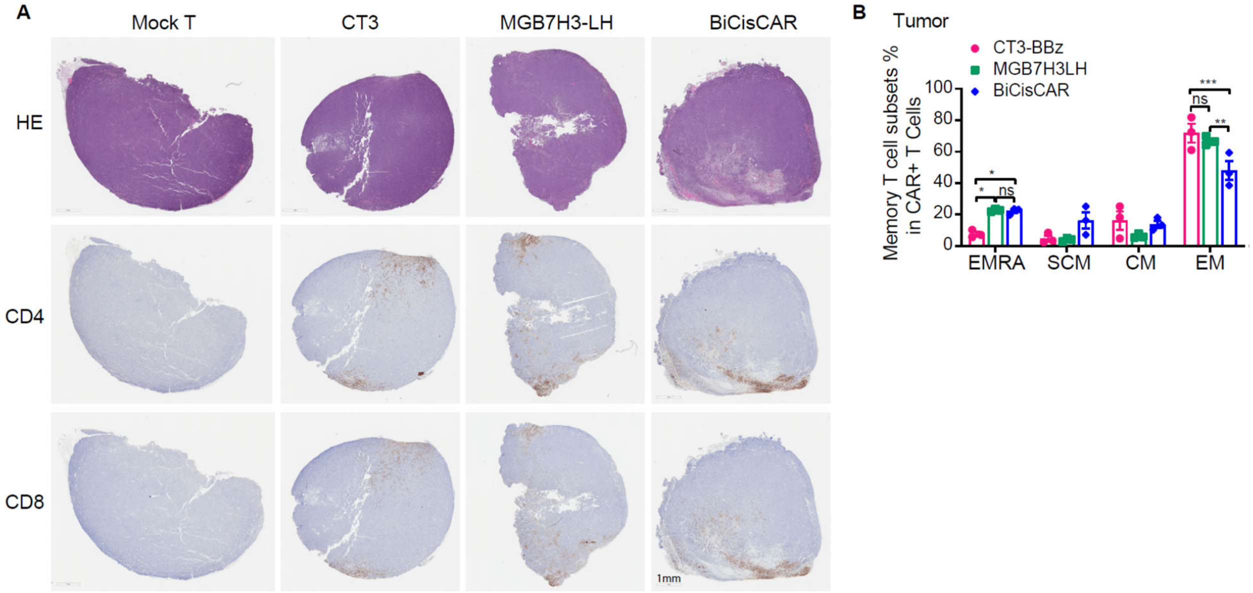

Supplemental Figure 10

T-cells infiltration pattern and memory T-cell phenotypes in tumors from NB PDX SJNB012407 subcutaneous model. (**A**) Representative low-power images of H&E and IHC staining within tumor xenografts after CAR T-cells infusion for 11 days. Tumors after Mock T-cells treatment contain rare, individualized T-cells. All CAR T-cells treated groups have focal, multifocal, or diffuse patterns of T-cell infiltration. Infiltrations is often at the tumor margins but intra-tumoral infiltration is seen for all groups. (**B**) The percentage of different memory T-cell states in CAR T-cells dissociated from tumors at day 28 post CAR T-cells infusion are shown. Data are shown as individual values and means ± SEM, $n = 3$ independent tumor from mice treated with indicated CAR T-cells. Statistical analysis performs with two-way ANOVA, Tukey's multiple comparisons test (*$p < 0.05$, **$p < 0.01$; ***$p < 0.001$; ****$p < 0.0001$; not significant, ns).

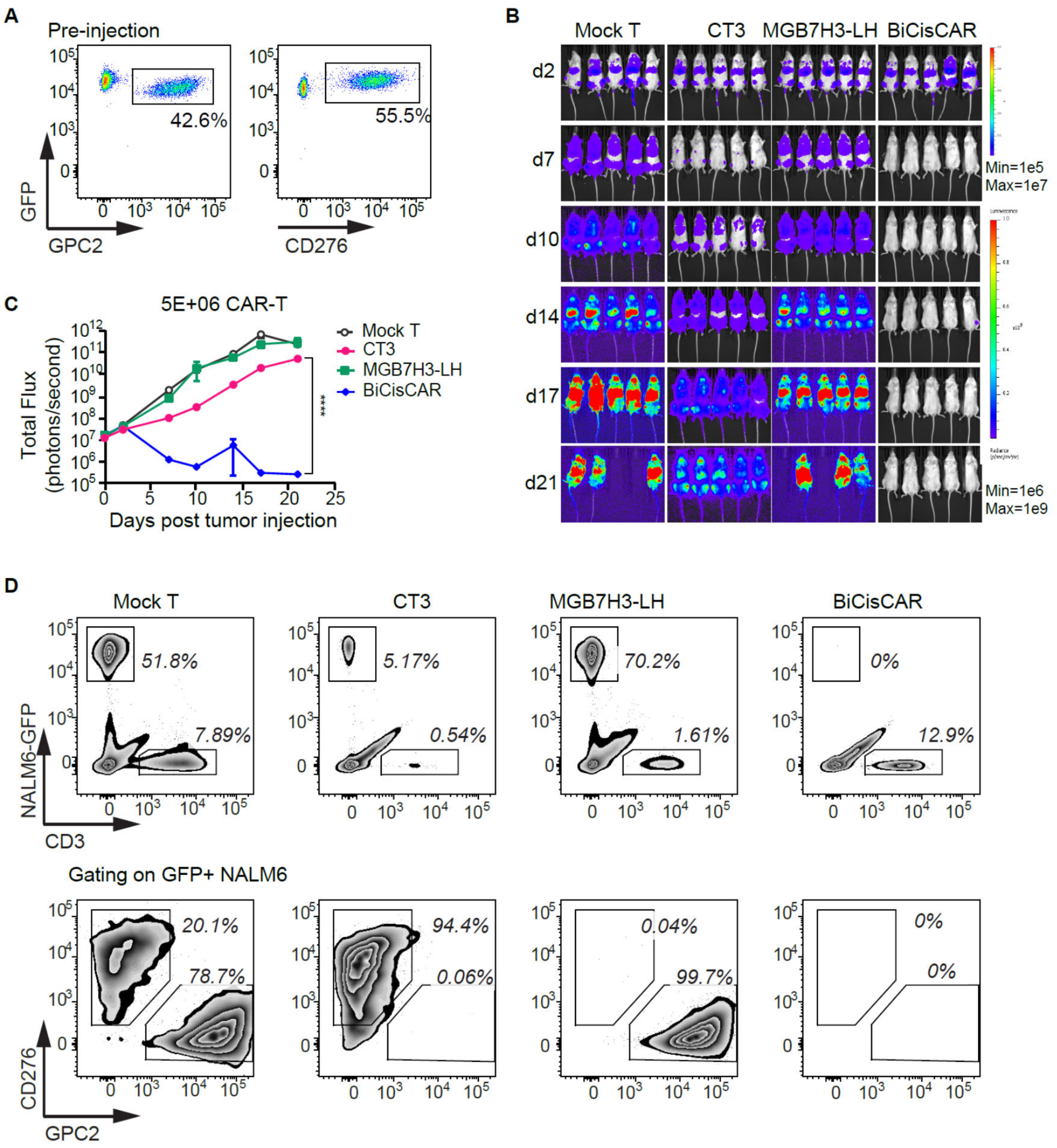

Supplemental Figure 11

BiCisCAR can effectively eliminate NALM6 leukemia cells expressing either GPC2 or CD276. (**A**) Representative flow-cytometric plots demonstrating the GPC2 and CD276 surface expression of 1:1 mixed leukemia before injection. (**B** and **C**) 5E6 CAR T-cells were infused into mice of 1:1 mixed NALM-GPC2 and NALM6-CD276 metastatic model on day 3 after tumor inoculation. Representative bioluminescence images (**B**) and bioluminescence kinetics (**C**) of NALM6 cells growth in the Leukemia metastatic model. Statistical analysis represents two-way repeated measures (RM) ANOVA (****$p < 0.0001$). (**D**) Representative flow cytometry plots of remaining NALM6-GPC2 or NALM6-CD276 cells in mice 21 days after 5E6 CAR T-cells infusion ($n = 5$).

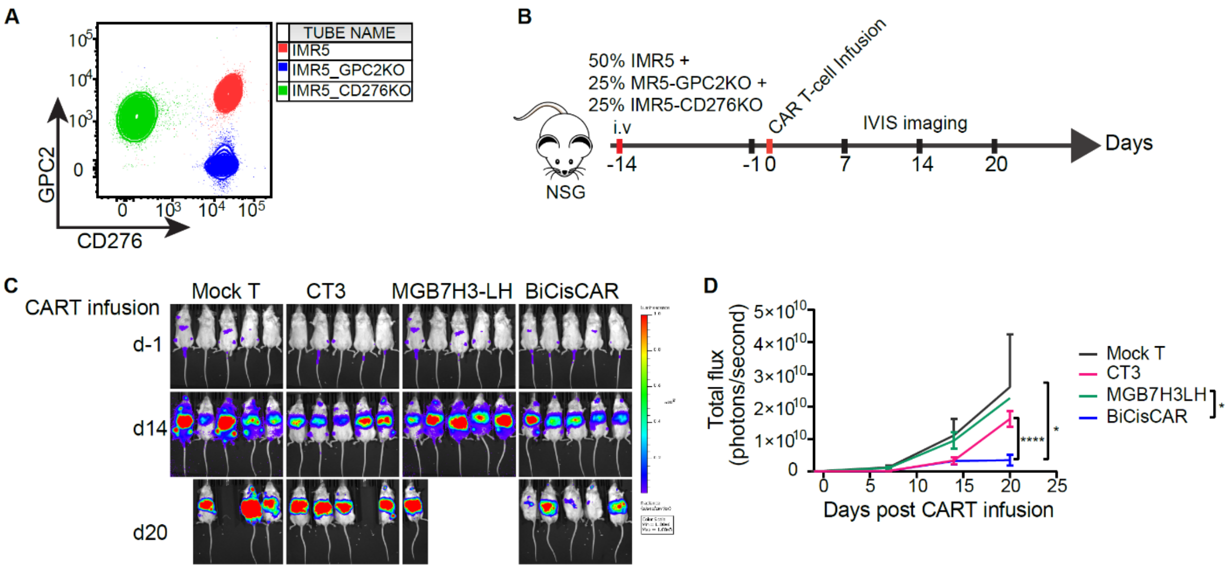

Supplemental Figure 12

BiCisCAR outperforms single CARs *in vivo* suppressing IMR5 cells heterogeneously expressing GPC2 and CD276. (**A**) Representative flow-cytometric plots demonstrating the GPC2 and CD276 surface expression in IMR5 (red), IMR5-GPC2KO (blue), or IMR5-CD276KO (green). (**B**) Schema of the heterogeneous neuroblastoma metastatic model infused with 2E6 CAR T-cells on day 14 after tumor inoculation. (**C** and **D**) Representative bioluminescence images (C) and bioluminescence kinetics (D) of IMR5 cells growth in the metastatic model before and post CAR T-cells treatment. Data are shown in means ± SEM, $n = 5$. Statistical analysis between MGB7H3LH and BiCisCAR before 14 days performed with two-way repeated measures ANOVA (*$p = 0.0241$). ****$p < 0.0001$ for CT3 versus BiCisCAR and *$p = 0.0366$ for Mock T versus BiCisCAR determined by mixed effects analysis due to the death of mice at day 21.

SUPPLEMENTAL TABLES

Supplemental Table 1

Summary of current on-going CAR T cell clinical trials for neuroblastoma.

Clinical Tiral #	Target	Location	status
NCT03721068	GD2	UNC Lineberger Comprehensive Cancer Center	Recruiting
NCT02311621	LA-CAM	Seattle Children's Hospital	Active, not recruiting
NCT04483778	CD276	Seattle Children's Hospital	Recruiting
NCT04897321	CD276	St. Jude Children's Research Hospital	Not yet recruiting
NCT04432649	CD276	Shenzhen Geno-Immune Medical Institute	Recruiting
NCT04864821	CD276	PersonGen BioTherapeutics (Suzhou) Co., Ltd.	Not yet recruiting
NCT04637503	CD276	Shenzhen Geno-Immune Medical Institute	Recruiting
NCT04691713	CD276	PersonGen BioTherapeutics (Suzhou) Co., Ltd.	Recruiting

Supplemental Table 2

CAR specific primers and probes used in ddPCR

Primers Name	Sequence
LH1_Fwd	CCGCCGTGCATTACTATGA
LH2_Fwd	TGGAACGACGTTGACTACTG
LH3_Fwd	AGGATATAGTGGCTACGATGGA
LH4_Fwd	GGGATATTGTAGTGGTGGTAGC
LH6_Fwd	GCGAGAGGTTACAGTTATGACG
LH7_Fwd	CGAGAGGTTACAGCTATGATGAC
CT3_Fwd	CCATCCACGTTCGGTACT
G27-HL_Fwd	AACAGACTTACAGTCCCCC
CartLH_FAM	CAACACCGGCGCCCACCATC
LH_common_Rev	ATGTAGATGTCACAGGCGAAG
m276HL_Fwd	GGAGTTACCATATGGACGTCTG
m276-HL_Rev	CAGGGTGGCTGGAGACT
m276LH_Fwd	TTATTACTGTCAGCAGCGTAGC
m276LH_Rev	GACCTTCACTGAGCTTCCAG
MGB7H3HL_Fwd	CAGAGGCCGGGAGAATATCTA
MGB7H3HL_Rev	AGGGGGACTGGGTCAGC
MGB7H3LH_Fwd	ACAACTACCCTTTCACCTTCG
MGB7H3LH_Rev	CGAAGCTGGAGAAGGTGAAG
h8H9_HL_Fwd	CACCCTGGTGACCGTGA
h8H9_HL_Rev	TTTCGCCTGGGCTCACA

h8H9_LH_Fwd	GCACCAAACTGGAACTGAAAG
h8H9_LH_Rev	CACCCAGTTAATATCATAGTTGGTAAAG
CartHLVL_FAM	TGGAGGTTCTGGTGGAGGTGGAT
GFP_Fwd	CTTCGCCCGCACCTTCA
GFP_Rev	GTTGCTGTGCAGCTCCT
GFP_FAM	CACATGCACTTCAAGAGCGCCATC
GFP_HEX	CACATGCACTTCAAGAGCGCCATC
Luc_Fwd	GCACATATCGAGGTGGACATTA
Luc_Rev	CCACGATCCGATGGTTTGTAT
Luc_FAM	AAGCTATGAAGCGCTATGGGCTGA
MKL2-lastExon_F	AGATCAGAAGGGTGAGAAGAATG
MKL2-lastExon_R	GGATGGTCTGGTAGTTGTAGTG
MKL2LastExon_HEX	TGTTCCTGCAACTGCAGATCCTGA
MKL2LastExon_FAM	TGTTCCTGCAACTGCAGATCCTGA

Supplemental Table 3

Cell hashing antibodies and antibody-oligo conjugates directed against T-cell antigens were used in multimodal assay

Hashtag Antibodies	Reactivity	Barcode Sequence
TotalSeq™-C0251 anti-human Hashtag 1 Antibody	Human	GTCAACTCTTTAGCG
TotalSeq™-C0253 anti-human Hashtag 3 Antibody	Human	TTCCGCCTCTCTTTG
TotalSeq™-C0254 anti-human Hashtag 4 Antibody	Human	AGTAAGTTCAGCGTA

CITE-seq Antibodies name	Surface marker	Isotype	Clone
TotalSeq™-C0072 anti-human CD4 Antibody	CD4	Mouse IgG1, κ	RPA-T4
TotalSeq™-C0046 anti-human CD8 Antibody	CD8α	Mouse IgG1, κ	RPA-T8
TotalSeq™-C0063 anti-human CD45RA Antibody	CD45RA	Mouse IgG2b, κ	HI100
TotalSeq™-C0087 anti-human CD45RO Antibody	CD45RO	Mouse IgG2a, κ	UCHL1
TotalSeq™-C0148 anti-human CD197 (CCR7) Antibody	CD197 (CCR7)	Mouse IgG2a, κ	G043H7
TotalSeq™-C0154 anti-human CD27 Antibody	CD27	Mouse IgG1, κ	O323
TotalSeq™-C0156 anti-human CD95 (Fas) Antibody	CD95	Mouse IgG1, κ	DX2
TotalSeq™-C0140 anti-human CD183 (CXCR3) Antibody	CD183 (CXCR3)	Mouse IgG1, κ	G025H7
TotalSeq™-C0144 anti-human CD185 (CXCR5) Antibody	CD185 (CXCR5)	Mouse IgG1, κ	J252D4
TotalSeq™-C0071 anti-human CD194 (CCR4) Antibody	CD194 (CCR4)	Mouse IgG1, κ	L291H4
TotalSeq™-C0143 anti-human CD196 (CCR6) Antibody	CD196 (CCR6)	Mouse IgG2b, κ	G034E3
TotalSeq™-C0390 anti-human CD127 (IL-7Rα) Antibody	CD127	Mouse IgG1, κ	A019D5
TotalSeq™-C0147 anti-human CD62L Antibody	CD62L(L-selection)	Mouse IgG1, κ	DREG-56
TotalSeq™-C0085 anti-human CD25 Antibody	CD25	Mouse IgG1, κ	BC96
TotalSeq™-C0386 anti-human CD28 Antibody	CD28	Mouse IgG1, κ	CD28.2

TotalSeq™-C0389 anti-human CD38 Antibody	CD38	Mouse IgG1, κ	HIT2
TotalSeq™-C0125 anti-human CD44 Antibody	CD44	Mouse IgG1, κ	BJ18
TotalSeq™-C0146 anti-human CD69 Antibody	CD69	Mouse IgG1, κ	FN50
TotalSeq™-C0355 anti-human CD137 (4-1BB) Antibody	CD137	Mouse IgG1, κ	4B4-1
TotalSeq™-C0171 anti-human/mouse/rat CD278 (ICOS) Antibody	ICOS	Armenian Hamster IgG	C398.4A
TotalSeq™-C0158 anti-human CD134 (OX40) Antibody	CD134(OX40)	Mouse IgG1, κ	Ber-ACT35 (ACT35)
TotalSeq™-C0088 anti-human CD279 (PD-1) Antibody	PD-1	Mouse IgG1, κ	EH12.2H7
TotalSeq™-C0169 anti-human CD366 (Tim-3) Antibody	TIM-3	Mouse IgG1, κ	F38-2E2
TotalSeq™-C0151 anti-human CD152 (CTLA-4) Antibody	CTLA-4	Mouse IgG2a, κ	BNI3
TotalSeq™-C0189 anti-human CD244 (2B4) Antibody	2B4(CD244)	Mouse IgG1, κ	C1.7
TotalSeq™-C0089 anti-human TIGIT (VSTM3) Antibody	TIGIT	Mouse IgG2a, κ	A15153G
TotalSeq™-C0155 anti-human CD107a (LAMP-1) Antibody	CD107a	Mouse IgG1, κ	H4A3
TotalSeq™-C0250 anti-mouse/human KLRG1 (MAFA) Antibody	KLRG-1	Syrian hamster IgG	2F1/KLRG1
TotalSeq™-C0090 Mouse IgG1, κ isotype Ctrl Antibody	Mouse IgG1, κ isotype	Mouse IgG1, κ	MOPC-21
TotalSeq™-C0091 Mouse IgG2a, κ isotype Ctrl Antibody	Mouse IgG2a, κ isotype	Mouse IgG2a, κ	MOPC-173
TotalSeq™-C0092 Mouse IgG2b, κ isotype Ctrl Antibody	Mouse IgG2b, κ isotype	Mouse IgG2b, κ	MPC-11

Supplemental Table 4

CAR binders specific primers used for enrichment library

	**Primers Names**	**primer seq 5'-3'**
Forward Primers	10X Patial Read1	CTACACGACGCTCTTCCGATCT
	10X Fwd SI	AATGATACGGCGACCACCGAGATCTACACTCTTTCCCTACACGACGCTC
Reverse Primers	CD3z-41BB_rev (outer primer)	GCTGAACTTCACTCTCAGTTCACATCC
	LH1-7_Rev_Adap	GTGACTGGAGTTCAGACGTGTGCTCTTCCGATCTCCGGATGAGGAGACGGTGA
	CT3_Rev_Adap	GTGACTGGAGTTCAGACGTGTGCTCTTCCGATCCGCTTTCAGCTCCAGCTTGGTC
	CD276-Rev_Adap	GTGACTGGAGTTCAGACGTGTGCTCTTCCGATCGCGTCGTGGTGGATCC
	G27-HL/m276-HL_Rev_Adap	GTGACTGGAGTTCAGACGTGTGCTCTTCCGATCCGTCGTGGTGGATCCTTTAATCT
	MGB7H3-HL_Rev_Adap	GTGACTGGAGTTCAGACGTGTGCTCTTCCGATCGTCGTGGTGGATCCCTTGATTTC
	h8H9-LH_Rev_Adap	GTGACTGGAGTTCAGACGTGTGCTCTTCCGATCGTCGTGGTGGATCCGCTGCT
	h8H9-HL_Rev_Adap	GTGACTGGAGTTCAGACGTGTGCTCTTCCGATCGTCGTGGTGGATCCTTTCAGTTC

Supplemental Table 5

Top 25 genes list exclusively defining CAR T-cells cluster 11 filtered by fold change > 2, comparing cluster 11 vs other CD8 clusters (0, 2, 4, 5, 6, 9 ,13) of CAR T-cells from the scRNA-seq data

gene	avg_log2FC	p_val	pct.1	pct.2	p_val_adj
CCL4	3.49417354	2.51E-38	0.33	0.052	8.43E-34
CCL3	3.17806952	6.16E-57	0.535	0.128	2.07E-52
CSF2	2.528728	7.39E-38	0.325	0.052	2.48E-33
LTA	2.5040861	1.86E-66	0.586	0.132	6.23E-62
MIR155HG	2.17128226	3.06E-126	0.901	0.279	1.03E-121
IFNG	2.03953176	4.01E-35	0.386	0.098	1.34E-30
XCL1	2.00722709	1.57E-61	0.484	0.078	5.27E-57
IER3	1.61202271	1.95E-74	0.766	0.271	6.54E-70
CCL1	1.52371895	7.77E-17	0.118	0.009	2.61E-12
ZBED2	1.51669289	4.79E-82	0.901	0.453	1.61E-77
CXCL8	1.51443507	1.74E-22	0.258	0.061	5.84E-18
CAVIN3	1.47067359	4.39E-75	0.851	0.424	1.47E-70
IL13	1.33413529	4.55E-21	0.202	0.036	1.52E-16
SNHG15	1.32691416	5.70E-69	0.928	0.614	1.91E-64
NFKBIA	1.2875604	1.16E-54	0.906	0.634	3.89E-50
RGS16	1.19258918	6.03E-59	0.448	0.06	2.02E-54
CYP1B1	1.17155554	1.92E-50	0.689	0.263	6.43E-46
CD70	1.16074762	1.79E-48	0.817	0.495	6.00E-44
DDIT4	1.1502705	5.05E-45	0.896	0.648	1.69E-40
TRAF1	1.12451631	1.16E-62	0.848	0.464	3.90E-58
PIM3	1.1016475	1.53E-45	0.704	0.352	5.14E-41
DUSP4	1.10120851	2.25E-49	0.892	0.573	7.56E-45
ZFAS1	1.0904653	6.08E-30	0.851	0.621	2.04E-25
GZMB	1.08282119	3.15E-40	0.983	0.991	1.06E-35
DUSP2	1.02896176	1.61E-39	0.472	0.14	5.39E-35

Supplemental Table 6

Top 20 differentially expressed genes (DEGs) in CT3 CAR T-cells compared with 7 other anti-GPC2 CARs from above DE gene set

gene	avg_log2FC	p_val	pct.1	pct.2	p_val_adj
IFNG	1.34195023	4.82E-12	0.29	0.109	8.41E-08
LTA	1.30470073	5.55E-15	0.555	0.296	9.68E-11
CCL3	0.87765702	2.82E-09	0.382	0.202	4.92E-05
XCL1	0.62914844	9.12E-07	0.286	0.156	0.01591
PIM3	0.62364218	4.55E-15	0.541	0.296	7.94E-11
IER3	0.58113674	1.36E-06	0.534	0.377	0.0238
MIR155HG	0.55222014	3.32E-11	0.686	0.462	5.80E-07
ZBED2	0.55161384	4.42E-12	0.714	0.533	7.71E-08
SNHG15	0.51638225	2.30E-14	0.714	0.497	4.01E-10
FABP5	0.47933234	4.27E-07	0.763	0.694	0.00745
IL13	0.47730203	0.01223	0.261	0.19	1
GZMB	0.45834679	1.71E-10	0.898	0.794	2.99E-06
CYP1B1	0.45612182	3.83E-07	0.516	0.341	0.00668
DUSP2	0.42982261	9.52E-09	0.378	0.201	0.00017
BATF3	0.40877906	6.75E-06	0.735	0.637	0.1178
BIRC3	0.4026606	4.88E-06	0.615	0.472	0.0851
NCL	0.38746635	1.60E-08	0.915	0.856	0.00028
IL2RA	0.38687705	4.35E-08	0.848	0.758	0.00076
MYC	0.38464216	1.42E-06	0.36	0.213	0.02474
RGS16	0.37819074	2.84E-07	0.332	0.173	0.00496

Supplemental Table7

14 upregulated expression genes with fold change > 2 in IMR5 stimulated MGB7H3-LH CAR T-cells compared with 5 other anti-CD276 CARs

gene	avg_log2FC	p_val	pct.1	pct.2	p_val_adj
CCL4	2.63908	0.78003	0.167	0.143	1
CCL3	2.14669	0.72403	0.333	0.333	1
MTRNR2L12	1.62949	0.1471	1	0.69	1
IFNG	1.53051	0.39659	0.333	0.214	1
CCL4L2	1.41504	0.0098	0.167	0	1
CCL3L1	1.39232	0.24951	0.167	0.048	1
LTB	1.38414	0.38416	0.5	0.452	1
S100A10	1.25376	0.01574	0.833	0.619	1
LCP1	1.12928	0.01881	1	0.524	1
CNN2	1.10692	0.0035	0.667	0.214	1
NEAT1	1.09366	0.13684	0.667	0.357	1
MT-ND2	1.05827	0.59442	0.833	0.905	1
RAB8A	1.01381	0.00793	0.667	0.19	1
NUTM2B-AS1	1	0.00031	0.833	0.167	1

SUPPLEMENTAL REFERENCES

51. Qin H, et al. Preclinical development of bivalent chimeric antigen receptors targeting both CD19 and CD22. Mol Ther Oncolytics. 2018;11:127–137.

52. Wei JS, et al. Clinically relevant cytotoxic immune cell signatures and clonal expansion of T-cell receptors in high-risk MYCN-not-amplified human neuroblastoma. Clin Cancer Res. 2018;24(22):5673–5684.

53. Smith T, et al. UMI-tools: modeling sequencing errors in Unique Molecular Identifiers to improve quantification accuracy. Genome Res. 2017;27(3):491–499.

54. Stuart T, et al. Comprehensive integration of single-cell data. Cell. 2019;177(7):1888–1902.

55. Hafemeister C, Satija R. Normalization and variance stabilization of single-cell RNA-seq data using regularized negative binomial regression. Genome Biol. 2019;20(1):296.

56. Mulè MP, et al. Normalizing and denoising protein expression data from droplet-based single cell profiling. Nat Commun. 2020;13(1):2099.

57. Kramer A, et al. Causal analysis approaches in Ingenuity Pathway Analysis. Bioinformatics. 2014;30(4):523–530.

58. Godec J, et al. Compendium of immune signatures identifies conserved and species-specific biology in response to inflammation. Immunity. 2016;44(1):194–206.
